# Anionic lipid vesicles have differential effects on the aggregation of early onset-associated α-synuclein missense mutants

**DOI:** 10.1016/j.jbc.2022.102565

**Published:** 2022-10-05

**Authors:** Kathryn J.C. Watt, Richard M. Meade, Robert J. Williams, Jody M. Mason

**Affiliations:** Department of Biology and Biochemistry, University of Bath, Claverton Down, United Kingdom

**Keywords:** peptide, amyloid aggregation, lipid vesicles, early onset Parkinson’s disease, DIV, days *in vitro*, DMEM, Dulbecco’s modified Eagle’s medium, DMPS, 1,2-dimyristoyl-*sn*-glycero-3-phospho-l-serine, PD, Parkinson’s disease, PI, propidium iodide, PS, phosphatidylserine, P/S, penicillin/streptomycin, αS, α-synuclein, SUV, small unilamellar vesicle, TEM, transmission electron microscopy, ThT, thioflavin T

## Abstract

α-synuclein (αS) is the key component of synucleinopathies such as Parkinson’s disease (PD), dementia with Lewy bodies, and multiple system atrophy. αS was first linked to PD through the identification of point mutations in the *SNCA* gene, causing single amino acid substitutions within αS and familial autosomal dominant forms of PD that profoundly accelerated disease onset by up to several decades. At least eight single-point mutations linked to familial PD (A30G/P, E46K, H50Q, G51D, and A53T/E/V) are located in proximity of the region preceding the non-β amyloid component (preNAC) region, strongly implicating its pathogenic role in αS-mediated cytotoxicity. Furthermore, lipids are known to be important for native αS function, where they play a key role in the regulation of synaptic vesicle docking to presynaptic membranes and dopamine transmission. However, the role of lipids in the function of mutant αS is unclear. Here, we studied αS aggregation properties of WT αS and five of the most predominant single-point missense mutants associated with early onset PD in the presence of anionic 1,2-dimyristoyl-*sn*-glycero-3-phospho-l-serine lipid vesicles. Our results highlight significant differences between aggregation rates, the number of aggregates produced, and overall fibril morphologies of WT αS and the A30P, E46K, H50Q, G51D, and A53T missense mutants in the presence of lipid vesicles. These findings have important implications regarding the interplay between the lipids required for αS function and the individual point mutations known to accelerate PD and related diseases.

α-synuclein (αS) is a key protein associated with synucleinopathies that include Parkinson’s disease (PD), dementia with Lewy bodies, and multiple system atrophy. αS was first linked to PD through the identification of missense mutations resulting in familial autosomal dominant forms (1). Approximately 5 to 15% of PD cases have a genetic link, with around 1 to 2% of cases resulting from mutations in the *SNCA* gene encoding αS (2, 3, 4, 5, 6, 7). To date, eight single-point mutants of αS have been identified: A30G (8), A30P (9), E46K (10), H50Q (11, 12), G51D (13, 14), A53E (15, 16), A53V (17), and A53T (1). There are significant differences between the Parkinsonism experienced by individuals with these mutations (Table 1) (18). These variations indicate that pathogenesis of PD occurs *via* different molecular mechanisms depending on the specific *SNCA* mutation, giving rise to a range of distinct clinical features and age of onset. The precise function that αS plays in the onset and pathogenesis of PD remains unclear; however, the central role of the protein provides an unequivocal link between PD and αS: (i) αS is the main protein component of Lewy bodies, the pathological hallmark of PD (19); (ii) αS aggregates are toxic (20); and (iii) αS mutants associated with early onset PD result in a profound effect that accelerates the disease by up to several decades (in the case of G51D, up to 40 years before sporadic PD onset) (14). Therefore, an improved understanding of native αS function and the mechanisms under which it misfolds in the disease state are vital to progressing understanding of PD pathology and ultimately the pathway toward improved diagnosis and treatment.

**Table 1:** Details of αS variants and key differences

αS variant	Age of onset (mean ± SD, range)	Clinical phenotype	Clinical features	Aggregation rate compared with WT		Fibril type (in the absence of lipids)	References
				With lipids	Without lipids		
WT	60–75 (sporadic)	Classic PD	Bradykinesia along with muscle rigidity, resting tremor, or postural instability. Other motor symptoms can include hypomimia, micrographia, shuffling gait, and freezing. Nonmotor symptoms can include autonomic dysfunction, cognitive abnormalities, sleep disorders, anosmia, and depression, and other behavioral changes. Good response to l-DOPA	—	—	Predominantly straight	(8, 48, 49, 74, 92)
A30G	58 ± 12 (36–80)	Dementia with Lewy bodies phenotype	Rest tremor at onset, good initial response to l-DOPA. Nonmotor symptoms including severe cognitive decline, RBD, hallucinations, orthostatic hypotension, and autonomic dysfunction	—	Similar	Straight	(8)
A30P	60 ± 11 (54–76)	Classic PD	Resting tremor at onset, progressive parkinsonism (bradykinesia, rigidity, tremor, and postural instability), good response to l-DOPA, progressive cognitive decline with mild dementia	Similar	NA (conflicting literature)	Straight	(8, 9, 48, 49, 74, 93)
E46K	52 ± 12 (28–81)	Dementia with Lewy bodies phenotype	Bradykinesia at onset, progressive parkinsonism, rapid and severe cognitive decline, dementia, visual hallucinations, transient response to l-DOPA. Sleep disturbances precede disease onset	Decreased	Increased	Twisted (with pitch of ∼43 nm)	(10, 48, 49, 94, 95, 96, 97)
H50Q	66 ± 6 (60, 71)	Classic PD	Resting tremor at onset, rapid progression of motor symptoms, cognitive decline, no autonomic dysfunction, and good response to l-DOPA	Decreased	Increased	Straight	(11, 12, 49, 50, 98)
G51D	39 ± 14 (19–69)	Severe PD, some have MSA features	Resting tremor at onset, rapid disease progression, motor fluctuations, pronounced cognitive decline, dementia, persistent visual hallucinations, autonomic dysfunction, psychiatric signs including anxiety and depression, mild/moderate response to l-DOPA	Decreased	Decreased	Predominantly straight	(13, 14, 49, 50, 98)
A53E	39 ± 14 (25–62)	Severe PD, some have MSA features	Numbness/clumsiness at onset, progressive parkinsonism, severe dyskinesia, psychiatric signs, including panic attacks, anxiety, and depression, no cognitive decline, good response to l-DOPA	—	Decreased	Straight (narrower than WT)	(15, 16, 49, 99, 100)
A53T	47 ± 7 (30–76)	Severe PD, some have MSA features	Variable symptoms at onset including muscle rigidity, postural instability, bradykinesia and rigidity, cognitive decline, and dementia; mostly good l-DOPA response. Resting tremor is less common	Increased	Increased	Predominantly twisted (with pitch of ∼100 nm)	(1, 48, 49, 74, 101, 102, 103)
A53V	56 ± 1 (55, 57)	Classic PD, some have psychosis or cognitive decline	Tremor at onset, slowly progressive parkinsonism, good response to l-DOPA, mild cognitive decline, and psychiatric disturbances	—	Increased	Straight	(17, 100)

Abbreviations: l-DOPA, levorotatory form of dihydroxyphenylalanine; MSA, multiple system atrophy; NA, not applicable; RBD, rapid eye movement sleep behavior disorder.

Shown are age of onset, clinical phenotype, clinical features, and fibril structures historically reported. Table adapted from Ruf *et al.* (18) and Whittaker *et al.* (104).

αS is a 140-amino acid protein that when studied *in vitro*, in the absence of lipid, has historically been considered intrinsically disordered and highly soluble (21). Natively, however, αS is predominantly found within presynaptic nerve terminals, where it abundantly colocalizes with lipids (22, 23). Although the native function of αS is not completely understood, it is thought to be involved with synaptic vesicle transport and fusion with the presynaptic membranes within dopaminergic neurons (23, 24, 25). When bound to lipid membranes, αS readily reconfigures from a natively unfolded (random coil) conformer to a structure rich in α-helical content (26, 27, 28). However, when located within Lewy bodies, αS is mainly found to have been converted into highly ordered β-sheet–rich amyloid aggregates (19, 29, 30, 31). These important structural distinctions, combined with the profound changes implemented by single amino acid substitutions associated with early onset PD, warrant further investigation into their effect upon aggregation in the presence of lipid vesicles.

Investigating the interactions between αS and lipid vesicles has led to the identification of novel aggregation pathways and fibril structures, and as a result, is a growing area of interest (27, 32, 33, 34, 35, 36, 37, 38, 39, 40). In particular, αS interacts with small unilamellar vesicles (SUVs) composed of anionic lipids (such as 1,2-dimyristoyl-*sn*-glycero-3-phospho-l-serine [DMPS]) *via* positively charged lysine residues in the N-terminal region of αS (41). Because of the high curvature and increased phospholipid packing defects, αS preferentially binds to SUVs (∼40 nm diameter). These are comparable in size to vesicles found at the synaptic terminal of dopaminergic neurons, relative to large unilamellar vesicles (>100 nm) (41, 42, 43). Moreover, αS binding to lipid vesicles can act as an anchor point for aggregation, accelerating primary nucleation, and this is particularly the case at lower lipid:protein ratios because of the sufficiently high concentration of monomeric αS remaining in solution to contribute to fibril growth (27, 32, 38). The role of DMPS is not fully understood; however, phosphatidylserine (PS) has been shown to be an abundant component of synaptic vesicle membranes (38, 44); the PS concentration levels increase by more than a third in individuals with incidental PD (45); and DMPS is associated with αS-facilitated synaptic vesicle docking and is linked to SNARE complex formation (46). Therefore, lipid vesicles composed of DMPS are a useful tool in further investigating the αS aggregation properties (27, 35, 47).

Although there is a much focus on αS oligomers, complimentary studies investigating fibrils and their polymorphs can also provide much insight into the misfolding and aggregation. For example, recent work has shown that WT αS can adopt a unique range of fibril polymorphs when incubated in the presence of lipid (DMPS) vesicles (Fig. 1) (35). Here, we sought to expand upon this finding by examining the aggregation pathway of six αS single-point variants known to significantly accelerate the disease pathology (WT, A30P, E46K, H50Q, G51D, and A53T). Aggregation was monitored in the presence of DMPS vesicles to probe primary nucleation events. Studies were also conducted in the absence of vesicles, with both undertaken for prolonged periods to elucidate their role in the misfolding and aggregation of early onset mutants. Determination of the structures of filamentous αS is helpful to further our understanding of how the structure and toxicity of aggregates is coupled, enabling the development of improved therapeutics and diagnostics.

**Figure 1:**
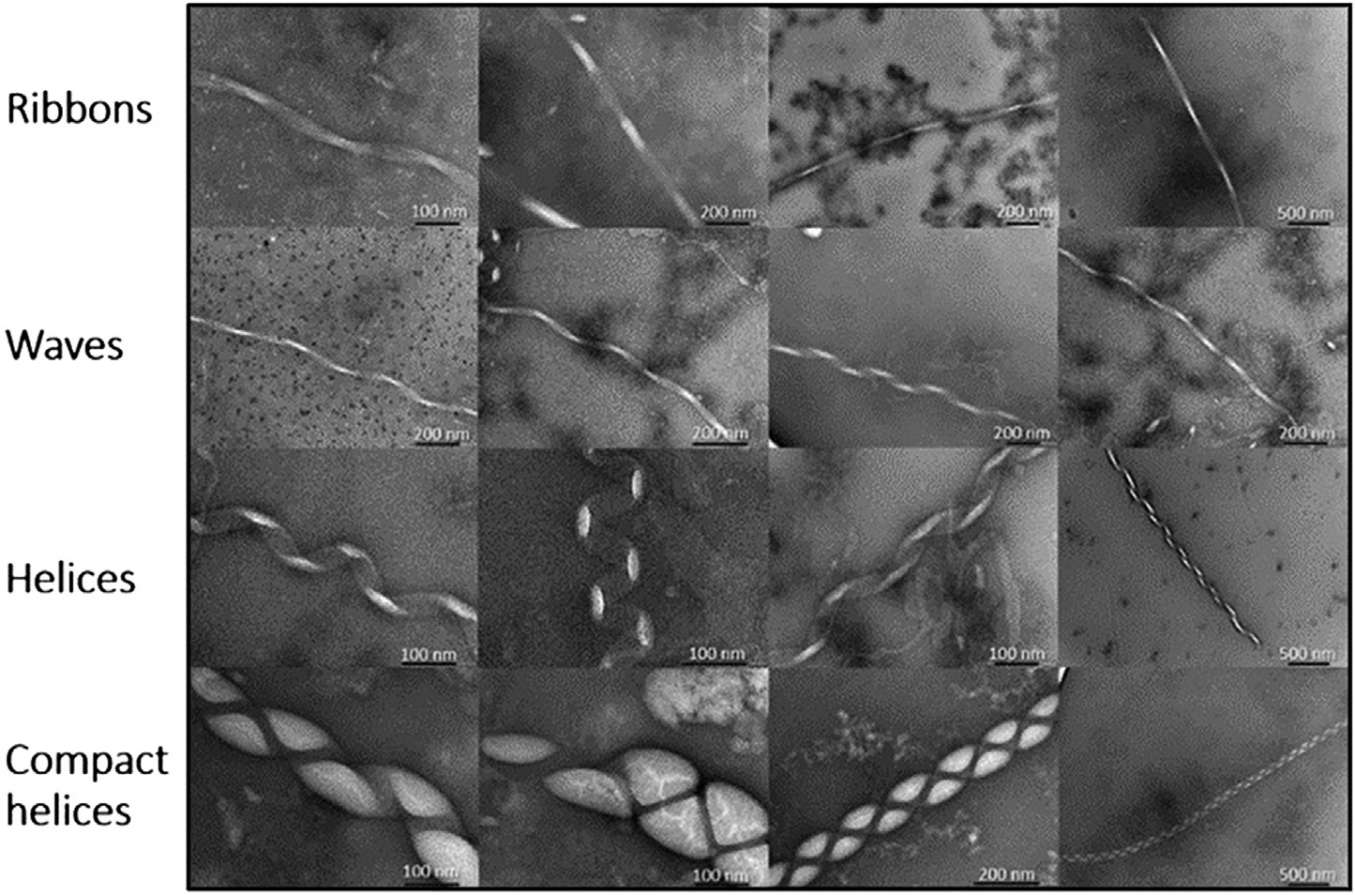
**A large variety of fibril polymorphs were formed upon incubation with DMPS lipid vesicles.** Previous work (32) demonstrated that large helical polymorph aggregates would self-assemble, with varying degrees of helicity, when αS (100 μM) was incubated in the presence of DMPS lipid vesicles (200 μM) at 37 °C for ∼200 h. Reproduced from Ref. (35). DMPS, 1,2-dimyristoyl-*sn*-glycero-3-phospho-l-serine; αS, α-synuclein.

## Results

There are a number of studies where effect of mutation upon αS aggregation has been investigated (48, 49, 50, 51, 52, 53, 54, 55). However, there is growing evidence that lipids play a significant role in both native αS function in synaptic transmission as well as within disease (40). Therefore, here, we study the role of lipid-induced aggregation to investigate the effect of point mutants upon the aggregation pathway, global secondary structure, and resulting fibril structures. Previous work identified a unique range of large αS fibril polymorphs, which formed under extended incubation times with DMPS SUVs (Fig. 1) (35). The structures were striking in both their large size, relative to those previously reported, and by their varying macromolecular helical content, from ribbons to wave-like helices of long pitch, to more compact and bulkier polymorphs. Of particular significance was that all previously reported fibril structures formed under similar conditions were done so in either the absence of lipid or with much shorter incubation periods and lower DMPS concentrations (51, 56, 57, 58, 59). By incubating αS with lipid vesicles, at a higher concentration, and greatly extending the incubation time, it was demonstrated that initial structures observed during early time points of the stationary phase were consistent with those previously reported in the literature. However, prolonged incubation within the stationary phase led to the formation of much larger and more mature helical fibril polymorphs. Here, we sought to build upon these findings, by expanding the study to include five of the most predominant and well-described single-point mutants associated with familial PD.

Six variants of αS (WT, A30P, E46K, H50Q, G51D, and A53T) were incubated with DMPS at 30 °C under quiescent conditions. The anionic lipid DMPS was chosen as a model lipid since αS preferentially binds to negatively charged phosphate groups (through interaction with the positively charged lysine residues of αS) (26, 40); PS has been shown to be an abundant component of synaptic vesicle membranes (38, 44); the PS concentration levels increase by more than a third in individuals with incidental PD (45); and DMPS is associated with αS-facilitated synaptic vesicle docking (46). Furthermore, we used an [αS:DMPS] ratio of 1:2 for our primary nucleation thioflavin T (ThT) assays, since this has been previously found to result in increased αS aggregation. In contrast, at significantly higher ratios, αS aggregation becomes inhibited (*i.e.*, at a ratio where αS predominantly exists in the lipid-bound α-helical state) (27). The concentration of αS in healthy neurons is estimated to be in the region of 70 to 140 μM; therefore, here we have utilized a concentration of 100 μM αS, to align with physiologically relevant concentrations (60).

In the following sections, we compare the lipid binding of mutants monitored by CD, followed by an overview comparison of the ThT aggregation profiles of all the αS mutants. There are a number of limitations to using ThT fluorescence to monitor αS aggregation. For example, ThT binds the surface grooves formed by the cross-β amyloid structure *via* hydrophobic or electrostatic interactions, resulting in relatively high or low fluorescence intensities (61, 62, 63). Therefore, variation in ThT intensity may be a result of the extent of fibril formation, or changes to fibril morphology, or the presence of other components. Moreover, ThT does not detect oligomers or protofibrils that form early in the aggregation pathway. Therefore, to support the ThT observations, we have also used CD and transmission electron microscopy (TEM). Herein, following a general overview of the ThT aggregation profiles, we discuss in more detail the individual αS mutants from ThT, CD, and EM datasets.

### Comparative lipid vesicle–binding studies monitored by CD

**Figure 2:**
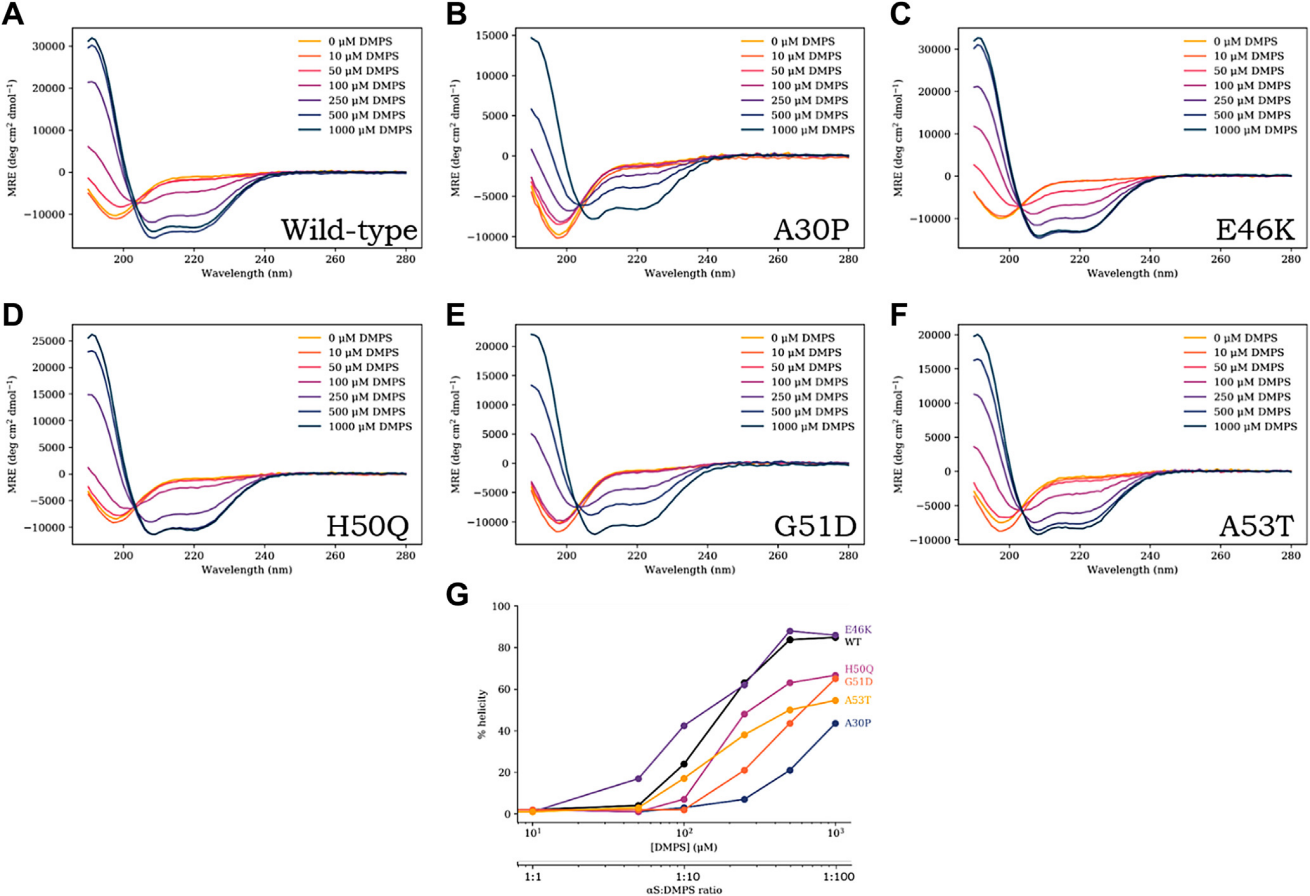
**αS mutants change from random coil to form α-helix in the presence of excess DMPS vesicles.***A*–*F*, WT, A30P, E46K, H50Q, G51D, and A53T, respectively. Shown are CD spectra collected at an αS concentration of 10 μM, with increasing concentration of DMPS from 0 μM (*yellow*) to 1000 μM (*dark blue*). In all cases, addition of DMPS results in a shift from random coil to an increasingly α-helical structure that varies in magnitude for each mutant depending on the precise ratio. *G*, percentage helicity for each of the αS mutants at increasing DMPS concentrations, determined using DichroWeb (77, 78). DMPS, 1,2-dimyristoyl-*sn*-glycero-3-phospho-l-serine; αS, α-synuclein.

αS readily reconfigures from a natively unfolded (random coil) conformer to a conformer rich in α-helical content when bound to DMPS lipid vesicles. Therefore, we first sought to determine if any of the single amino acid substitutions result in changes to lipid-binding properties by measuring the CD of fully monomerized αS (10 μM) in the presence of increasing concentrations of DMPS vesicles (0–1000 μM) (Figs. 2 and S10). By plotting the percentage helicity *versus* DMPS concentration for each of the αS variants, we determined that E46K adopts a higher α-helical content at lower αS:DMPS stoichiometries relative to WT (Fig. 2*G*). Consistent with the literature, it was observed that A30P and, interestingly, G51D adopt lower α-helical signatures in the presence of DMPS vesicles relative to WT even at high lipid concentrations, with neither reaching a plateau of α-helical content at the highest ratio of 1:100 (αS [10 μM]:DMPS [1000 μM]) (64, 65). In particular, A30P required the highest DMPS concentration (1000 μM, 1:100) before any helical signature was observed (Fig. 2*B*), whereas E46K adopted a comparable helical signature at a αS:DMPS 10 times lower (100 μM DMPS; 1:10; Fig. 2*C*). This highlights the significant differences that single-point mutations confer on the ability of αS to bind to lipid vesicles and consequently to adopt helical signatures.

### Comparative ThT monitored lipid-induced aggregation

**Figure 3:**
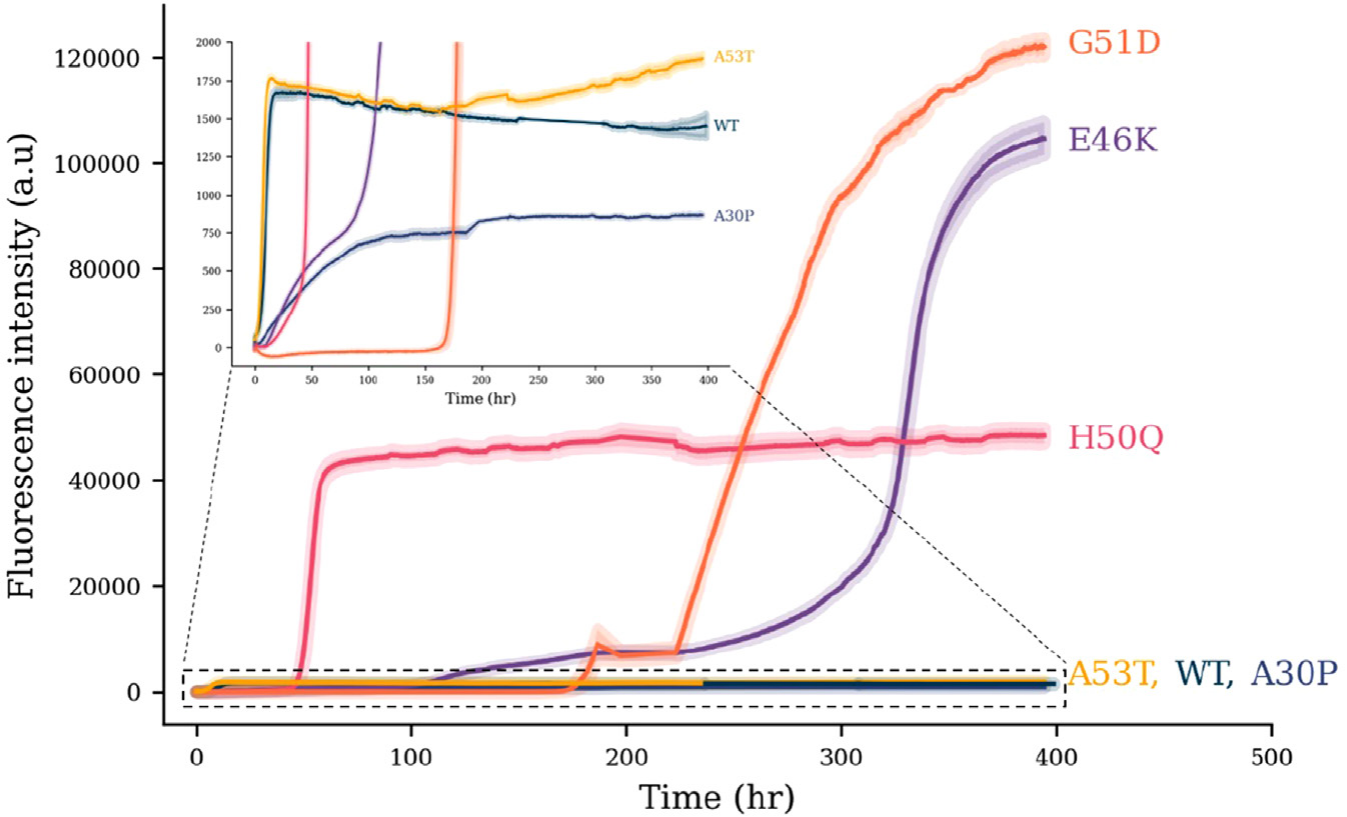
**ThT aggregation profiles for WT αS and early onset mutants in the presence of DPMS vesicles.** Respective αS mutant (100 μM), DMPS vesicles (200 μM), and ThT (50 μM) were incubated in 20 mM sodium phosphate buffer (pH 6.5) at 30 °C under quiescent conditions. The average of three repeats is shown with the standard error. Profiles display stark differences in ThT intensity between G51D (*orange*), E46K (*purple*), and H50Q (*pink*) compared with WT (*dark blue*), A30P (*blue*), and A53T (*yellow*). *Inset* is zoomed to show additional detail for WT, A30P, and A53T. DMPS, 1,2-dimyristoyl-*sn*-glycero-3-phospho-l-serine; αS, α-synuclein; ThT, thioflavin T.

ThT aggregation assays (Fig. 3) were used to monitor the effect of single amino acid substitutions on the rate of lipid-induced primary nucleation of αS (Figs. S11 and S13). During the initial stages of aggregation, our findings are in agreement with those previously published using the same approach of aggregation in the presence of DMPS (51). However, we significantly lengthened the incubation time once the stationary phase (previously considered the experimental end point) was reached in order to see how this would affect the formation of larger polymorphs. In most cases, the stationary plateau remained uniform. However, in several instances (E46K, G51D, and H50Q), there were significant further increases in ThT fluorescence intensity, beyond the assumed stationary phase. In all cases, these changes only occurred after prolonged incubation times (tens to hundreds of hours). This was most notable for G51D, which remained unchanged for ∼200 h, before undergoing a large increase in ThT intensity (midpoint at 271 h). In addition, E46K underwent a further large increase in ThT intensity after ∼300 h (midpoints at 140 and 330 h). Specific results for each construct are discussed later and summarized in Table 2. All values shown are averaged from at least three experimental repeats. In the following sections, we discuss each mutant from ThT, CD, and EM datasets.

**Table 2:** Summary of ThT, CD, and TEM data

αS variant	ThT midpoint time (h) (105)	ThT midpoint time increase (relative to WT) (h)	ThT height (% relative to WT) (Fig. 3)				Secondary structural content (% β-sheet) (Figure 4, Figure 5, Figure 6, Figure 7, Figure 8, Figure 9*B*)				Median fibril width (nm) (see also EM data (Figure 4, Figure 5, Figure 6, Figure 7, Figure 8, Figure 9)			Fibril description		Kinetic changes compared with other studies	
			40 h	100 h	200 h	400 h	0 h	200 h	400 h	600 h	200 h	400 h	600 h	200 h	400/600 h	With lipids	Without lipids
WT	10	0	0	0	0	0	9	13	15	19	12	15	15	Mature fibrils	Mature fibrils	↑	=
A30P	37	+27	−78	−56	−50	−20	10	16	12	14	23	16	15	Protofibrils	Mature fibrils	↓	NA (conflicting literature)
E46K	28	+18	−72	−25	+373	+7100	13	12	31	18	21	16	15	Mature fibrils	Mature fibrils	↑	↓
H50Q	52	+42	−78	+2734	+3012	+3232	10	17	20	26	10	12	12	Mature fibrils	Mature fibrils	=	↓
G51D	289	+271	−102	−101	+362	+8290	10	10	32	31	—	20	15	Protofibrils	Mature fibrils	NA	↓
A53T	6	−4	+3	+2	+4	+30	12	15	15	26	11	14	18	Mature fibrils	Mature fibrils	=	=

Abbreviation: NA, not applicable.

ThT midpoint (to first stationary phase) values were determined using the half-time plotter from AmyloFit (105). The absolute value is shown, along with the relative change from WT (in hours). ThT intensity is also shown as the percentage relative to WT for the time points: 40, 100, 20, and 400 h. Secondary structural content was determined using DichroWeb (77, 78). Shown is the total β-sheet content for the time points: 0, 200, 400, and 600 h. TEM data were analyzed using ImageJ (79). Shown are the calculated median values for the measured fibril widths at time points: 200, 400, and 600 h and brief description of the predominant fibril morphology observed. Kinetic changes compared with other studies (with lipids or without lipids where ↑ indicates the rate reported herein is greater, ↓ is decreased, or = is the same as previously reported in the literature).

### WT

**Figure 4:**
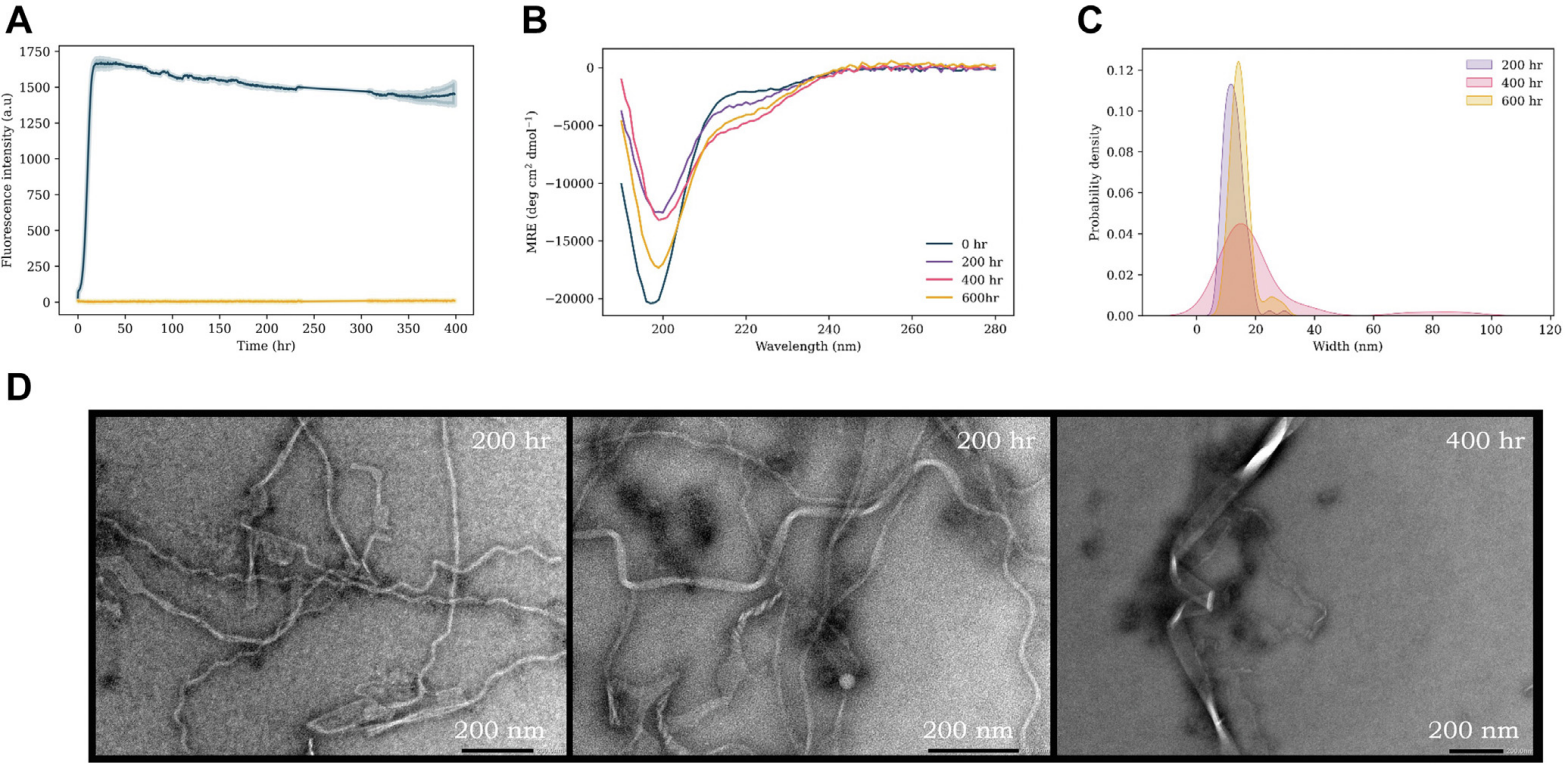
**WT αS aggregation experiments.***A*, ThT kinetics of WT (100 μM) aggregation with DMPS vesicles (200 μM, *blue*) and without vesicles (*yellow*) in 20 mM sodium phosphate buffer (pH 6.5) at 30 °C under quiescent conditions. The average of three repeats is shown with the standard error. *B*, CD spectra of WT + DMPS vesicles at increasing time points throughout the aggregation pathway: 0 h (*blue*), 200 h (*purple*), 400 h (*pink*), and 600 h (*yellow*). *C*, probability density plot of the range of widths (nanometer) (measured with ImageJ) of fibrils observed. *D*, TEM images of WT samples with lipids throughout the aggregation pathway. DMPS, 1,2-dimyristoyl-*sn*-glycero-3-phospho-l-serine; αS, α-synuclein; TEM, transmission electron microscopy; ThT, thioflavin T.

The aggregation kinetics and fibril formation of WT αS have previously been extensively studied. Here, our findings replicated those described in previous work and were comparable to previous studies carried out under similar primary nucleation conditions (Fig. 4*A*) (35, 51). Starting from monomer, a plateau was reached after ∼12 h, after which the ThT intensity remained constant. However, CD measurements (Fig. 4*B*) undertaken throughout the aggregation pathway demonstrate that global random coil signal is lost, while a slight β-sheet character increases over time (Fig. S1—characterized by a loss of signal at ∼190 nm and a gain at ∼218 nm). Interestingly, this occurred despite ThT kinetic experiments indicating a plateau at the stationary phase, therefore suggesting aggregation was complete. The same sample was analyzed by TEM at three time points over the aggregation pathway (Fig. 4*D*) to provide a direct visualization of the fibril types becoming populated. At 200 h, we observed ribbon and wave polymorph with widths of ∼12 nm, and pitches of ∼75 to 90 nm, similar to our previous reports and those of others under these conditions (35). With prolonged incubation times, we observed that these mature into more complex structures of the same overall broad topology. For instance, some fibrils were observed to increase to an average width of ∼15 nm, with fibrils of 20 to 40 nm widths and even 70 to 90 nm observed (Fig. 4*C*). The pitch of these fibrils also varied widely from ∼50 to 300 nm.

### A30P

**Figure 5:**
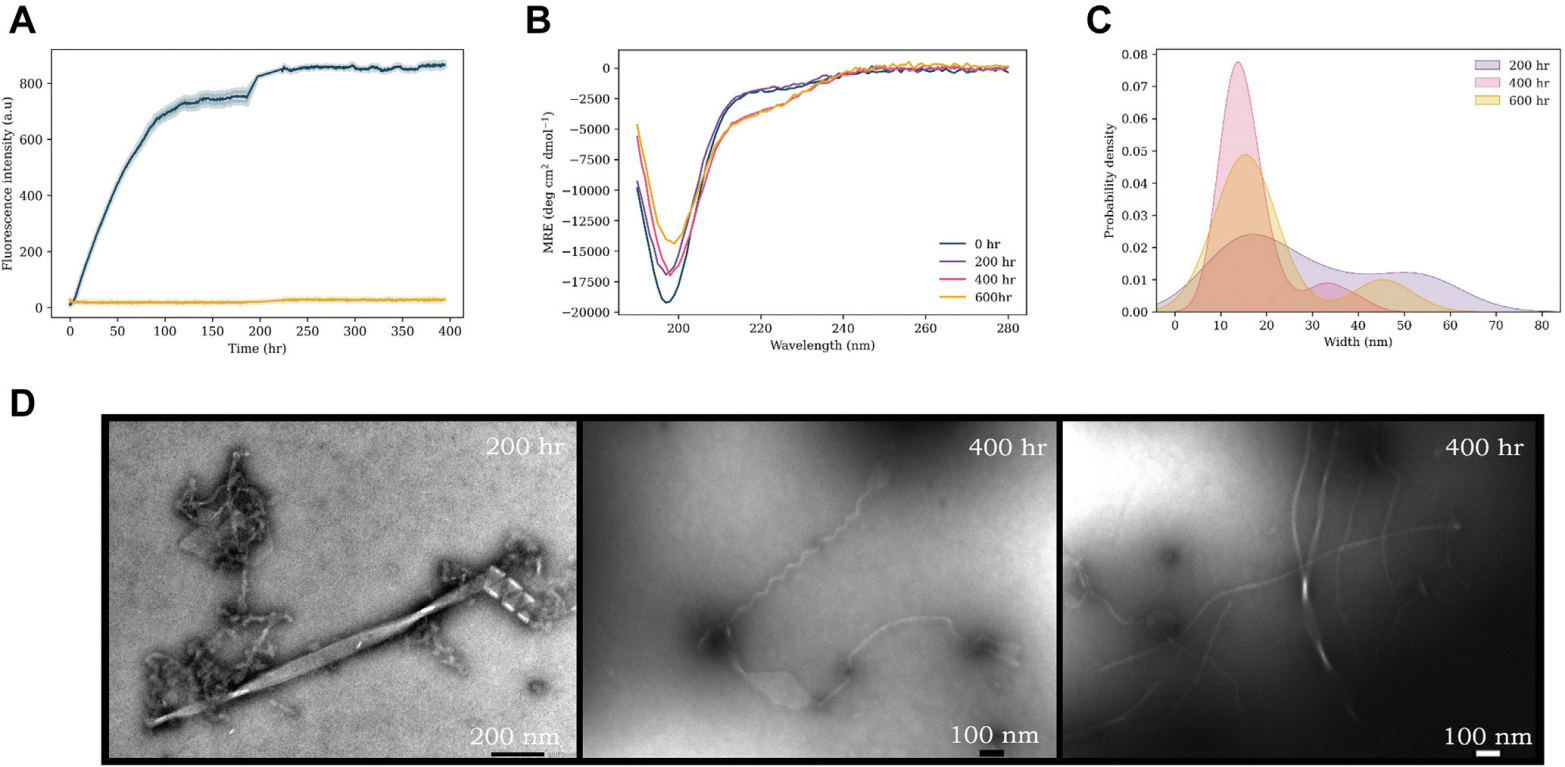
**A30P αS aggregation experiments.***A*, ThT kinetics of A30P (100 μM) aggregation with DMPS vesicles (200 μM, *blue*) and without vesicles (*yellow*) in 20 mM sodium phosphate buffer (pH 6.5) at 30 °C under quiescent conditions. The average of three repeats is shown with the standard error. *B*, CD spectra of A30P + DMPS vesicles at increasing time points throughout the aggregation pathway: 0 h (*blue*), 200 h (*purple*), 400 h (*pink*), and 600 h (*yellow*). *C*, probability density plot of the range of widths (nanometer) (measured with ImageJ) of fibrils observed. *D*, TEM images of A30P sample with lipids at various time points throughout the aggregation pathway. DMPS, 1,2-dimyristoyl-*sn*-glycero-3-phospho-l-serine; αS, α-synuclein; TEM, transmission electron microscopy; ThT, thioflavin T.

A30P is the only mutation studied here that is located outside the preNAC interface between protofilaments published within solid-state NMR and cryo-EM structures (56, 57, 58, 59). Of all the αS mutants, the behavior and kinetics of A30P is the one with the least consensus to date: there are reports of both increased and deceased aggregation rates, whereas others report rates consistent with WT αS (51, 52, 66, 67, 68, 69). However, in the presence of lipid vesicles, Flagmeier *et al.* (51) found that A30P had a slightly enhanced aggregation rate, and that the fibrils formed had a very similar morphology, compared with WT. Here, we use ThT monitored kinetics and fibril formation for A30P in the presence of lipid vesicles (Fig. 5). Surprisingly, we find that in contrast to WT, A30P displays no observable lag phase. Despite this, the time taken to reach stationary phase was longer (approximately 120 h *versus* 20 h), with the final intensity at the stationary phase approximately half that of WT (Fig. 5*A*). To ensure that this observation was not artifactual, the experiment was repeated on multiple occasions with different protein preparations and broadly similar patterns of increase in ThT intensity observed on all occasions. The lower ThT intensity indicates a reduced extent of β-sheet formation. This may reflect a change in the aggregation mechanism between A30P and WT. However, CD spectra (Fig. 5*B*) collected at increasing time points in the aggregation pathway show that a large extent of random-coil secondary structure is conserved even with long incubation times. After extended incubation, a modest increase in β-sheet signal was observed but was lower in magnitude than that of other mutants (Fig. S1). Morphology of fibrils was analyzed at increasing time points using TEM (Fig. 5*D*). Relative to WT, at the 200 h time point, the predominant species were protofibrils of varying sizes and form. However, larger fibril polymorphs were also observed with widths varying between ∼10 to 25 nm and increasing to 45 to 60 nm (Fig. 5*C*), with pitches varying between 70 to 90 nm and up to ∼400 to 800 nm. With prolonged incubation, mature fibril structures were the predominant species observed. The fibrils displayed an average width of 15 nm, but fibrils up to 35 to 40 nm also observed. Ribbon and wave polymorphs were observed with pitches varying from 60 to 85 nm to ∼200 to 300 nm (Fig. 5*D*).

### E46K

**Figure 6:**
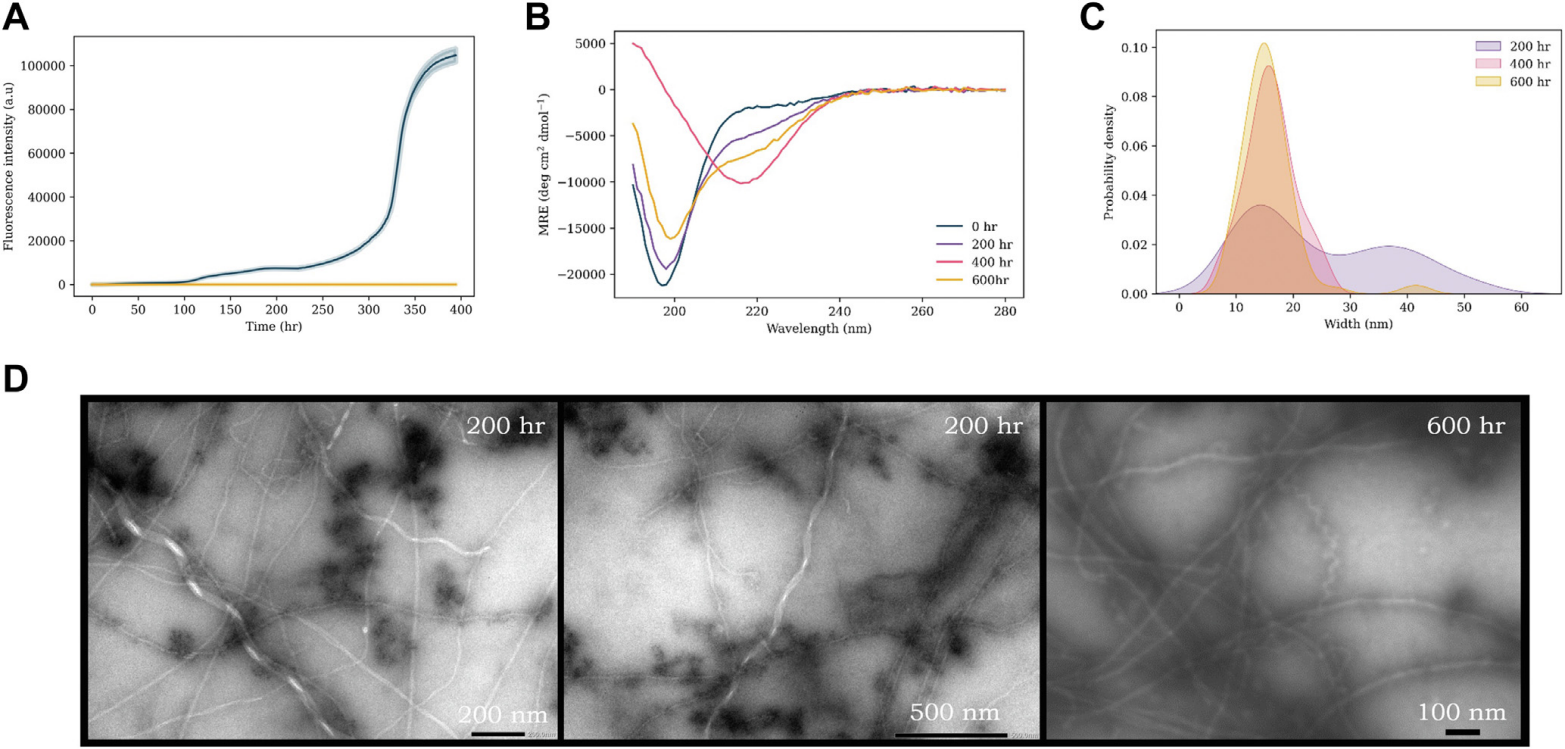
**E46K αS aggregation experiments.***A*, ThT kinetics of E46K (100 μM) aggregation with DMPS vesicles (200 μM, *blue*) and without vesicles (*yellow*) in 20 mM sodium phosphate buffer (pH 6.5) at 30 °C under quiescent conditions. The average of three repeats is shown with the standard error. *B*, CD spectra of E46K + DMPS vesicles at increasing time points throughout the aggregation pathway: 0 h (*blue*), 200 h (*purple*), 400 h (*pink*), and 600 h (*yellow*). *C*, probability density plot of the range of widths (nanometer) (measured with ImageJ) of fibrils observed. *D*, TEM images of E46K sample with lipids at increasing time points throughout the aggregation pathway. DMPS, 1,2-dimyristoyl-*sn*-glycero-3-phospho-l-serine; αS, α-synuclein; TEM, transmission electron microscopy; ThT, thioflavin T.

Many previous reports on E46K describe an accelerated aggregation rate relative to WT (48, 49, 70, 71, 72). However, in the presence of lipid vesicles, Flagmeier *et al.* (51) found the rate of E46K aggregation to be significantly reduced compared with WT, and fibrils were not observed. We also observed that the initial phase of aggregation is very much reduced relative to WT (Fig. 6*A*). However, after a significant period, a second phase of aggregation is detected in which the ThT intensity increases to ∼7500 (∼4× greater than for WT). Moreover, a third phase of E46K aggregation was observed after prolonged incubation in which the ThT intensity of E46K rises by another order of magnitude to >100,000 (50× greater than for WT). To ensure that this observation was not artifactual, the experiment was repeated on two further occasions with different protein preparations, with broadly similar patterns of dramatic increase in ThT intensity at prolonged incubation times observed on both occasions (Fig. S2). This significant increase in signal intensity, which up to that point appears as a stationary phase, is also reflected within the CD spectra (Fig. 6*B*). Whilst E46K initially presents as a random-coil structure (as for all αS variants), the extent of β-sheet character increases notably after 200 h, and by 400 h, the extent of β-sheet character is substantially increased (Figs. 6*B* and S1). Of all early onset mutants studied, E46K displays the greatest extent of β-sheet character at this time point, suggesting an alternative mode of aggregation.

Finally, TEM (Fig. 6*D*) was used to evaluate the fibril morphology at increasing time points during the aggregation pathway. At the 200 h time point, the majority of observed fibrils were straight with a width centered around 10 to 15 nm. However, also observed were fibrils containing helices and waves, of increased width and varying from ∼30 to 50 nm with pitches in the region of 200 to 350 nm. At prolonged incubation times (Fig. 6*D*), similar morphologies were continually observed with the majority of the snapshots containing straighter ribbon-like fibrils with an average width of 15 to 20 nm (Fig. 6*C*). Wave polymorphs were also observed with similar widths and pitches in the region of 55 to 300 nm (Fig. 6*D*).

### H50Q

Previous reports of the aggregation of H50Q have found that the rate is accelerated relative to WT (18, 55). However, in the presence of lipid vesicles, Flagmeier *et al.* (51) found that H50Q resulted in a significant reduction in the rate of lipid-induced aggregation compared with WT. Moreover, they reported that H50Q undergoes a second phase of aggregation after approximately 70 h with the resulting fibrils displaying a morphology similar to that of mature WT fibrils aggregated in the absence of lipid vesicles.

**Figure 7:**
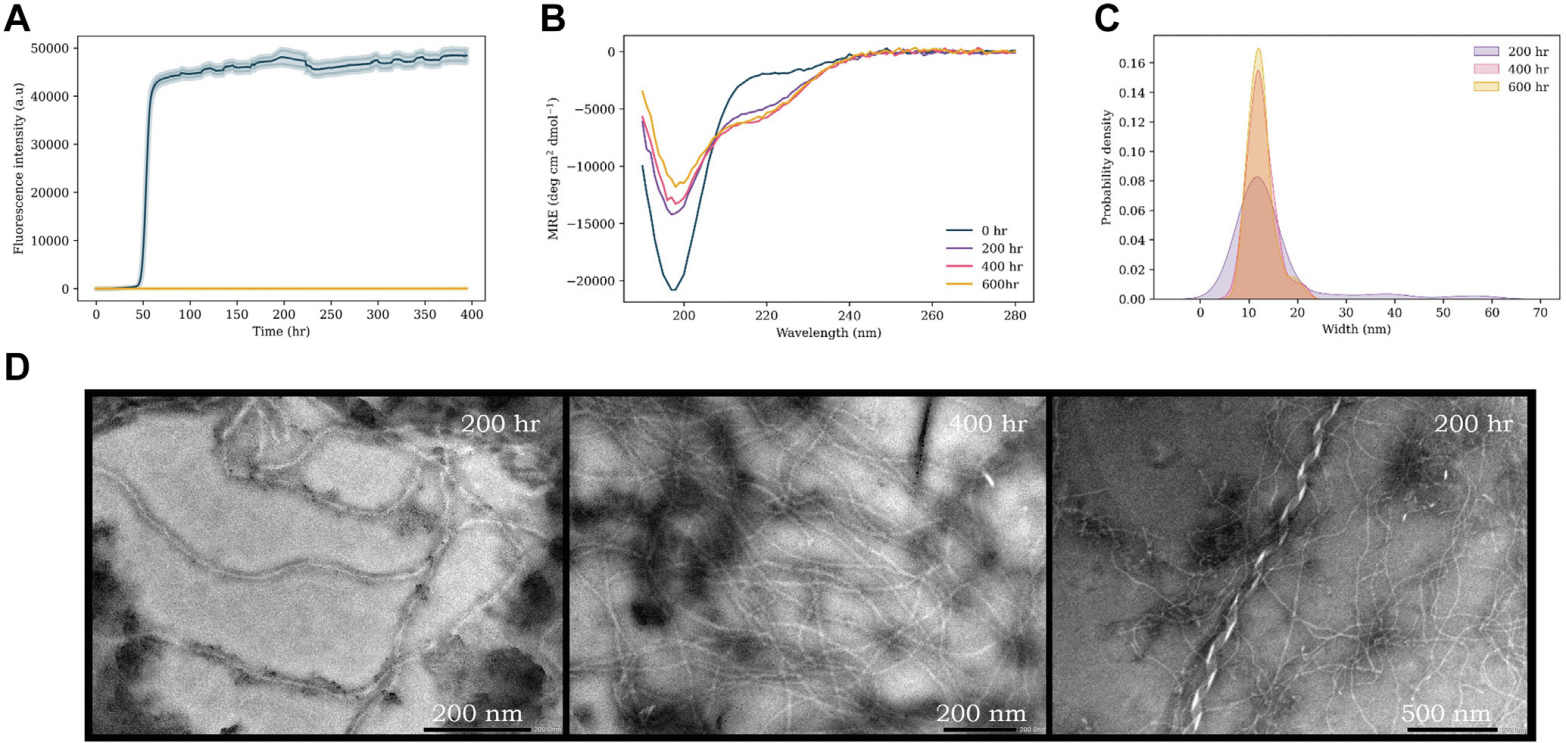
**H50Q αS aggregation experiments.***A*, ThT kinetics of H50Q (100 μM) aggregation with DMPS vesicles (200 μM, *blue*) and without vesicles (*yellow*) in 20 mM sodium phosphate buffer (pH 6.5) at 30 °C under quiescent conditions. The average of three repeats is shown with the standard error. *B*, CD spectra of H50Q + DMPS vesicles at increasing time points throughout the aggregation pathway: 0 h (*blue*), 200 h (*purple*), 400 h (*pink*), and 600 h (*yellow*). *C*, probability density plot of the range of widths (nanometer) (measured with ImageJ) of fibrils observed. *D*, TEM images of H50Q sample with lipids at various time points throughout the aggregation pathway. DMPS, 1,2-dimyristoyl-*sn*-glycero-3-phospho-l-serine; αS, α-synuclein; TEM, transmission electron microscopy; ThT, thioflavin T.

As shown in Figure 7, ThT kinetics for H50Q display a delayed aggregation midpoint relative to WT (52 h *versus* 10 h). Moreover, a sharp increase in ThT intensity was observed, approximately one order of magnitude higher than observed for WT (48,000 *versus* 2000). To ensure that this observation was not artifactual, the experiment was repeated on multiple occasions with different protein preparations, and broadly similar patterns of increase in ThT intensity were observed on all occasions. CD was used to monitor the secondary structure of H50Q (Fig. 7*B*) across the aggregation pathway: it was observed that the substantial increase in ThT fluorescence intensity was reflected in a significant change in the extent of β-sheet character of the protein. H50Q was found to contain the highest β-sheet intensity (Fig. S1) of any of the mutants at ∼200 h. Using TEM (Fig. 7*D*) to analyze the morphology of the H50Q fibrils at the various time points, it was observed that at 200 h, fibrils displayed an average width of 5 to 10 nm, which increased to 10 to 15 nm at 400 h (Fig. 7*C*). At all time points, the majority of the fibrils were of a meandering ribbon type, with no distinct waves or helices polymorphs. However, at 200 h, helical fibril polymorphs were observed of ∼100 nm width with a pitch ranging from 250 to 300 nm (Fig. 7*D*), highlighting that these were possible to produce in this mutation.

### G51D

**Figure 8:**
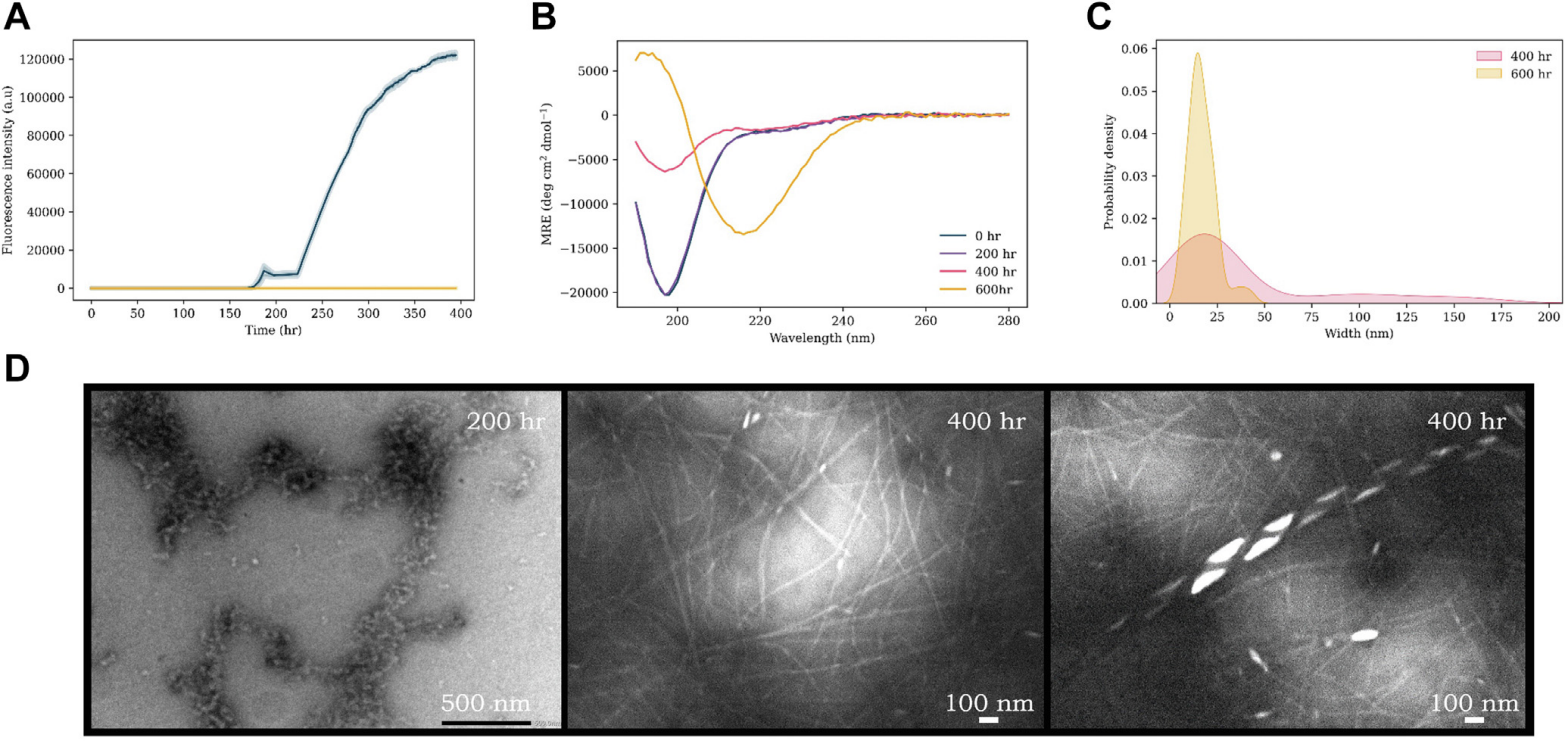
**G51D αS aggregation experiments.***A*, ThT kinetics of G51D (100 μM) aggregation with DMPS vesicles (200 μM, *blue*) and without vesicles (*yellow*) in 20 mM sodium phosphate buffer (pH 6.5) at 30 °C under quiescent conditions. The average of three repeats is shown with the standard error. *B*, CD spectra of G51D + DMPS vesicles at increasing time points throughout the aggregation pathway: 0 h (*blue*), 200 h (*purple*), 400 h (*pink*), and 600 h (*yellow*). *C*, probability density plot of the range of widths (nanometer) (measured with ImageJ) of fibrils observed. *D*, TEM images of G51D sample with lipids at various time points throughout the aggregation pathway. DMPS, 1,2-dimyristoyl-*sn*-glycero-3-phospho-l-serine; αS, α-synuclein; TEM, transmission electron microscopy; ThT, thioflavin T.

All previous reports of G51D aggregation have found it to aggregate at a decreased rate relative to WT, including in the presence of lipid vesicles where fibrils under these conditions have not previously been visualized (51, 73). We found ThT kinetics of G51D aggregation displayed a very long lag phase (significantly longer than for WT) in which there was no observable increase in ThT fluorescence intensity (Fig. 8*A*; 165 h *versus* 10 h). However, after approximately 165 h, the intensity of ThT fluorescence increased dramatically to approximately 60 times that of WT (120,000 *versus* 2000 AU). This is the first reported case of G51D aggregating to this extent. An order of magnitude difference in ThT intensity suggests either a much greater extent of β-sheet formation or that ThT is binding to the protein in a different way. Using CD spectroscopy (Fig. 8*B*), we observed that while there was a slight increase in percentage of β-sheet character at 200 and 400 h, there is a much greater increase by 600 h, suggesting an alternative form of aggregation. G51D resulted in the largest extent of β-sheet character relative to any other variant and reassuringly was reproducible across both experimental replicates and on multiple occasions (Figs. 8*A* and S2). The fibril morphology was visualized using TEM (Fig. 8, *C* and *D*). Initially, small oligomer/protofibrils were observed, but by 400 h, fibrils had formed. The fibrils observed displayed average widths of ∼15 to 20 nm and were mostly straight—relatively similar to those observed for E46K and H50Q, both of which also had at least an order of magnitude greater ThT intensity relative to WT. However, there was evidence of twisting within fibril strands at 600 h, and prominent helical fibril polymorphs were observed at 400 h of ∼9 μm in length, ∼300 nm pitch, and ∼100 to 175 nm in width (Fig. 8*D*).

### A53T

**Figure 9:**
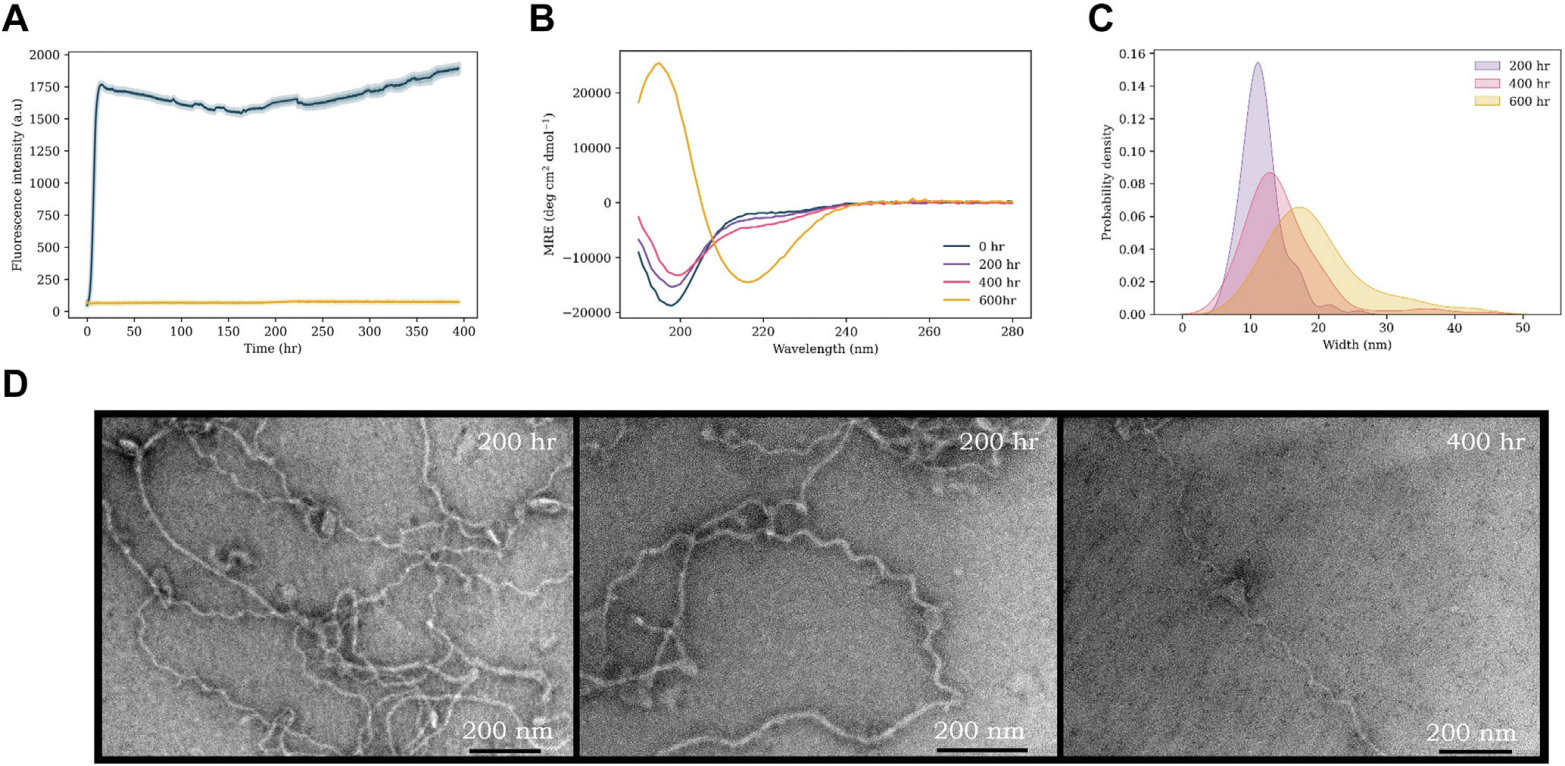
**A53T αS aggregation experiments.***A*, ThT kinetics of A53T (100 μM) aggregation with DMPS vesicles (200 μM, *blue*) and without vesicles (*yellow*) in 20 mM sodium phosphate buffer (pH 6.5) at 30 °C under quiescent conditions. The average of three repeats is shown with the standard error. *B*, CD spectra of A53T + DMPS vesicles at increasing time points throughout the aggregation pathway: 0 h (*blue*), 200 h (*purple*), 400 h (*pink*), and 600 h (*yellow*). *C*, probability density plot of the range of widths (nanometer) (measured with ImageJ) of fibrils observed. *D*, TEM images of A53T sample with lipids at increasing time points throughout the aggregation pathway). DMPS, 1,2-dimyristoyl-*sn*-glycero-3-phospho-l-serine; αS, α-synuclein; TEM, transmission electron microscopy; ThT, thioflavin T.

A53T is generally considered to aggregate at a (significantly) accelerated rate relative to WT, including in the presence of lipid vesicles, where Flagmeier *et al.* found that the fibrils formed had a very similar morphology to WT (51, 52, 53, 66, 74). In the presence of lipids, the ThT kinetics of A53T aggregation are in agreement with that reported in literature (Fig. 9*A*): the aggregation profile is similar to WT but with a shorter lag phase. A53T reaches its plateau after approximately 10 h, with the ThT fluorescence intensity remaining constant for the remaining incubation period. Analysis of the secondary structure by CD (Fig. 9*B*) was similar to the overall trend of other αS samples: a gradual decrease in random coil, with a proportionate increase in β-sheet character. It has been noted that the similar ThT intensities result in different levels of β-sheet character. This strongly indicates that the samples do not remain “stationary,” even within the stationary phase when the ThT fluorescence intensity has plateaued. This is supported by additional evidence from TEM, in which it is observed that the fibrils continue to change morphology with time within the ThT stationary phase: the average width increases from ∼10 nm to 15 nm to 20 nm (Fig. 9*C*). The fibrils observed for A53T were similar to those observed for WT and are also closest to WT relative to all other αS variants studied.

### Comparative cell toxicity studies monitored by propidium iodide Hoechst assay

**Figure 10:**
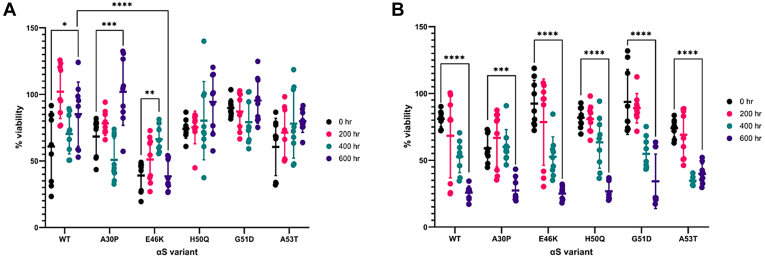
**Determination of cytotoxicity of the αS fibrils formed in the presence of lipid vesicles.***A*, αS (100 μM) with DMPS lipid vesicles (200 μM) and (*B*) αS (100 μM) were incubated under quiescent conditions at 30 °C for 0 hr (*black*), 200 hr (*pink*), 400 hr (*green*) or 600 hr (*purple*). To SH-SY5Y cell cultures, 20 μM monomer equivalents of these aged samples was added and incubated for 48 h before cell viability was measured by a PI/Hoechst assay. The percent viability corresponds to the live/dead ratio of each sample, which are normalized to the respective buffer solutions, representing the 100% value. The results are the mean of three independent treatments ±SD. ∗*p* < 0.05, ∗∗*p* < 0.01, ∗∗∗*p* < 0.001, and ∗∗∗∗*p* < 0.0001; two-way ANOVA followed by Tukey’s multiple comparisons test. DMPS, 1,2-dimyristoyl-*sn*-glycero-3-phospho-l-serine; PI, propidium iodide; αS, α-synuclein.

To assess the cytotoxicity of the αS variant fibril structures, an assay using SH-SY5Y cells differentiated into a neuronal phenotype was developed. Similar to the previously described ThT primary nucleation assays, αS (100 μM) and DMPS vesicles (200 μM) were incubated under quiescent conditions at 30 °C for the specified length of time (0, 200, 400, or 600 h, in sodium phosphate buffer [20 mM, pH 6.5]). Following incubation, 20 μM monomer equivalents of the αS solutions were added to the cell cultures. The cells were incubated for 48 h before viability was measured using a propidium iodide (PI) Hoechst assay. As shown in Figure 10*A*, an overall trend is observed that the cell toxicity reduces with increased incubation; however, the extent of this varies markedly between the αS variants. For example, the toxicity of G51D remains mostly consistent around 12%, and the reduction in A53T toxicity is not significant. On the other hand, despite fluctuations at each of the time points, the trend in reduced toxicity with increased incubation for WT and A30P is significant. Overall, this is consistent with low-n oligomers contributing to an increased toxicity profile, the trend observed for these mutants is that the extent of cell viability increases with increased sample age, indicating that the fibril structures formed under these conditions are increasingly less toxic to cells. Moreover, the viability of each of the αS variants is similar, except for E46K, which consistently was more toxic. This may correlate to E46K more readily binding to the lipid vesicles (Fig. 2).

Conversely, in the absence of lipid vesicles (Fig. 10*B*), the trend observed is reversed with each of the αS variants increasing in toxicity with increased incubation. The samples incubated in the absence of lipid vesicles did not result in a measurable ThT signal; therefore, it is assumed that these samples result in the formation of various oligomer species over the course of the assay but not measurable fibril species.

### Seeding potential of E46K αS fibril polymorphs

The presence of two distinct ThT transitions in the ThT kinetic curves raised the question of the different seeding potentials, with monomeric αS, of the fibril polymorphs produced. A preliminary investigation of this was performed for E46K αS, as this mutant presented the clearest distinct transitions. These results are presented in the supporting information (Fig. S12) and suggest that the initial fibrils produced from the lipids, showing a “wave” morphology, or the larger helical polymorphs, are unsuccessful as seeding material for further fibrillation in the absence of lipid, but that the fibrils produced after the second transition, which represent a classic fibril appearance, do seed the aggregation of monomeric αS. This presents an interesting property of the fibrils produced and warrants further investigation.

## Discussion

All eight currently identified missense mutations in the *SNCA* gene result in single-point mutations located in proximity of the preNAC region of αS. In particular, this region is known to bind to lipid membranes and is found to adopt an α-helical structure under these conditions (75, 76). In contrast, the C-terminal region (residues 98–140) has been reported to remain predominantly unstructured when αS is bound to lipid vesicles (75). Therefore, it has been hypothesized that some early onset mutants may function by changing the binding affinity of αS to lipid membranes, for example, by disrupting the α-helix formation, or increasing the propensity of the proteins to form toxic oligomeric conformers, and imposing a profound effect upon the native function of the protein. Whilst the amount of research investigating the pathophysiology of αS oligomers is growing, complimentary studies investigating αS fibril polymorphism can generate insight into misfolding and aggregation of αS, including potential correlations between αS fibril structure and maturation, and the resulting PD subtype. Ultimately, this could lead to the identification of new structural targets for the development of drugs to inhibit αS aggregation and fibril formation.

**Figure 11:**
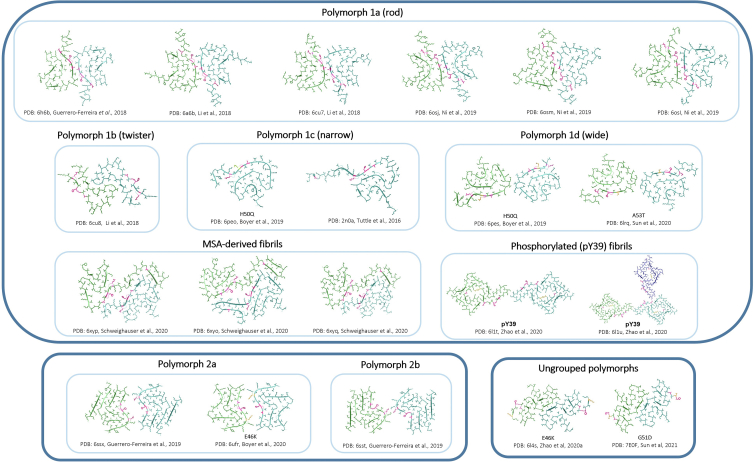
**αS fibril structures.** Currently known fibril structures of αS grouped by core similarity. Polymorph 1 have a Greek-key core, with the subgroups (a, b, c, and d) divided based on the residues involved at the interface. Most of the structures are for WT αS; however, there are two structures of E46K (polymorph 2a and ungrouped polymorph), two structures for H50Q (polymorph 1c and 1d), one structure for G51D (ungrouped polymorph), and one structure for A53T (polymorph 1d). Positions of known mutations are highlighted in *pink*. For the mutant structures, the mutation is highlighted in *orange* (56, 57, 58, 59, 81, 82, 83, 84, 85, 86, 87, 88, 89). Further structures are pending (90, 91). αS, α-synuclein.

In currently published fibril structures of αS, the point mutations studied are commonly located within the protein–protein interface (Fig. 11) (56, 57, 58, 59), again suggesting that these side chains are highly relevant to function and/or misfunction of αS. Of all currently known point mutations, E46K has the most profound effect on the structure of the fibril core: the salt bridge formed between E46 and K80 in polymorph 1 (Fig. 11) appears vital in stabilizing the compact Greek-key motif, and therefore, loss of this salt bridge in the E46K mutant destabilizes this motif, resulting in population of alternative polymorphs (polymorph 2a and ungrouped polymorph, Fig. 11) being formed. Other mutations (H50Q, G51D, and A53T) result in a decreased hydrophobicity and an increase in steric bulk but do not appear to fundamentally prohibit Greek-key motif formation. Rather, they impact upon precise side-chain packing preferences at the interface. For instance, atomic structures of H50Q and A53T (Fig. 11, polymorph 1d) fibrils confirm the core Greek-key motif; however, the interface between the protofilaments differs from a hydrophobic zipper (involving residues H50–E57) to an electrostatic interaction (involving T59 and K60). This electrostatic two-residue interface might be expected to be less stable than that of the hydrophobic zipper of the rod (1a) polymorph. Therefore, it can be hypothesized that the fibril structures of the point mutations exist in a more dynamic equilibrium, which could potentially lead to greater toxicity. Conversely, the versatility of the possible polymorphs is highlighted through the fibril structure of G51D, which was determined to not have the same Greek-key core but a core more similar to that of the ungrouped E46K structure (Fig. 11, ungrouped polymorphs). Therefore, the existence of at least three distinct polymorph groupings is clear, but which of these (if any) is biologically relevant, or if there are more possible polymorphs, remains unanswered.

Here, we report that five of the most prevalent and well-described mutations associated with familial PD form fibrils of the same type of helical polymorphs that we have previously reported upon incubation with lipids (Fig. 1) (35). We show that these helical polymorphs are not unique to WT αS and form with relative frequency amongst the familial mutants. In obtaining fibrils, the described polymorphs are formed under comparatively mild conditions (100 μM, 30 °C, quiescent conditions, long incubation times) relative to many methods reported (*e.g.*, much higher concentrations, vigorous shaking). Whilst we have not determined atomic-resolution morphologies of the fibril cores reported herein, the range of fibril widths observed suggest that a variety of protofilament interactions/interfaces are forming. The variety in overall gross morphology of the fibrils (*e.g.*, width and pitch) suggests that they contain more protofilaments per strand (since they are typically wider than the commonly occurring 10 nm wide fibrils containing two protofilaments) and may therefore be forming alternative packing arrangements, as indicated by the changes to the fibril pitch length. The population density of the fibers and their maturity (*e.g.*, how compact the helices were) varied between mutants, providing evidence for gross morphological preference of each mutant to form a given polymorph. For example, A53T reached stationary phase most rapidly; however, the helical polymorphs were the least mature of those observed. In contrast, the longer incubation time required to form fibrils for H50Q and G51D may ultimately lead to more mature structures (*e.g.*, polymorph 2 *versus* polymorph 1). The variation in fibril widths observed may also represent conversion between polymorph subgroups, with the most stable forming over longer time courses. The fibril cores within gross morphologies reported herein may be represented by a previously published structure but may also represent a new polymorph(s) and will require further exploration. Furthermore, the biological relevance of these fibrils is yet to be determined. Increasing our knowledge on the precise structures formed during aggregation holds significant promise in developing improved therapeutics tailored to specific epitopes that can modulate diseases in which αS presents.

## Conclusion

We investigated aggregation and subsequent fibril formation of WT αS and five of the most predominant mutants associated with familial PD. In particular, we probed the aggregation kinetics when αS is coincubated with lipid vesicles for a prolonged incubation period. In the majority of previous αS aggregation studies, including those in the presence of lipid vesicles, the end point is taken to be a point shortly after ThT intensity has plateaued, at which point no further changes to the αS species in equilibrium are thought to occur. Here, we report through CD and TEM that the fibril morphology and protein secondary structure continue to change despite no change to ThT intensity, highlighting the need to rely on other complementary techniques in measuring amyloidosis. Furthermore, in the case of E46K, a subsequent substantial increase in ThT intensity was observed after prolonged incubation beyond the stationary phase. This suggests that many αS reports are over too short a time frame, especially when considering the fibril morphology and the biological relevance to these studies.

Our results demonstrate that αS aggregation character can be divided into distinct groupings. For instance, WT and A53T display similar aggregation pathways (with A53T aggregation significantly accelerated) and similar fibril structures throughout the pathway. End-point fibril structures for E46K, H50Q, and G51D were all comparable, being narrower and straighter relative to WT. These samples also displayed at least an order of magnitude greater ThT intensity at the end point than WT; but the pathway in which these samples aggregated varied considerably. A30P did not display much resemblance to the other mutants in that the ThT intensity remained relatively low, as did the β-sheet signal according to CD; a few mature fibril structures were observed, with the prominent species being smaller oligomeric or protofibrilar structures that did not display the same level of ordered morphology as observed with the other αS variants. However, for every mutant investigated, a range of fibril polymorphs of a similar type to the WT helical polymorphs previously reported, were observed. This highlights that these polymorphs are not unique to WT αS but can also be formed by at least five (A30P, E46K, H50Q, G51D, and A53T) of the eight αS mutants associated with familial PD. These could in time be used to improve understanding of the role of these mutations upon the disease pathway. For example, do lag times and/or end fibril morphology correlate to disease onset, severity, or symptoms developed? However, a united model that describes the relationship between lipid binding, aggregation pathway, fibril morphology, and disease onset, progression, and symptoms for these mutants is still currently unknown. Further work will be required to understand if the fibril structures reported within have any resemblance to those identified *in vivo* and hence could provide further evidence of the associated between αS and lipid vesicles within the disease pathway.

## Experimental procedures

### αS expression and purification

Full-length (1–140) αS was recombinantly expressed in BL21(DE3) *Escherichia coli* cells using a pET21a plasmid (WT αS plasmid was a gift from the Michael J. Fox Foundation, Addgene plasmid #51486). Subsequent early onset mutants were created using site-directed mutagenesis using the WT gene. Overnight cultures (2XYT, 10 ml) of the transformed *E. coli* were used to inoculate 2XYT (1 l) cultures containing ampicillin (100 mgl^−1^), which were grown (37 °C, 200 rpm) to an absorbance of 0.6 to 0.8 at 600 nm. Protein expression was induced with IPTG (final concentration of 1 mM), and cells were harvested by centrifugation (5000 rpm, 20 min, 4 °C) following incubation (37 °C, 200 rpm, 4 h). The bacterial cell pellet was resuspended in 20 mM Tris buffer (pH 8) with 1 cOmplete protease inhibitor tablet (Roche). Following freeze–thawing at −20 °C, the cells were lysed by sonication. The soluble fraction of the lysate was separated from the cell debris by centrifugation (20,000 rpm, 20 min, 4 °C) and boiled (95 °C, 10 min) to precipitate impurities (αS remains soluble). Precipitated proteins were discarded after centrifugation (18,500*g*, 20 min, 4 °C), and ammonium sulfate was added to the supernatant to create a 30% solution, which was gently agitated (room temperature, 1 h) to precipitate the protein (including αS). The precipitated protein was collected by centrifugation (18,500*g*, 20 min, 4 °C) and resuspended with gentle agitation in 20 mM Tris buffer (pH 8, 4 °C). The protein was purified by anionic exchange chromatography on an ÄKTA pure purification system (GE Healthcare) with a 5 ml HiTrap Q HP (GE Healthcare) prepacked column. Fractions containing the purified protein were combined and further purified by size-exclusion chromatography using a HiLoad 16/60 Superdex 75 pg (GE Healthcare) prepacked column and buffered exchanged into the experimental buffer (20 mM sodium phosphate buffer [pH 6.5]). αS eluted between 54 and 64 ml. The protein was aliquoted and flash frozen in liquid nitrogen and stored at −80 °C until required.

Concentration of purified αS was determined by UV (280 nm) using a 2 mm quartz cuvette and an excitation coefficient of 4836 M^−1^ cm^−1^. Purity of the eluted protein was confirmed by SDS-PAGE, and the correct product was confirmed *via* mass spectroscopy using an Agilent QTOF (ESI-QTOF) mass spectrometer. CD spectral scan was used to confirm that the monomeric stock solutions of αS were random coil (Figs. S3–S8).

### Preparation of lipid (DMPS) vesicles

Suspension of DMPS (sodium salt) in 20 mM sodium phosphate buffer (pH 6.5, 2 mM) was incubated on a Thermomixer compact (Eppendorf) shaker (45 °C, 1400 rpm, 3 h). The solution frozen and thawed five times using dry ice (15 min) and a Thermomixer compact (Eppendorf) shaker at 45 °C (0 rpm, 5 min). Lipid vesicles of the desired size were formed *via* sonication (Soniprep 150 plus sonicator, amplitude 10, 5 × 30 s with 30 s rest between rounds). Vesicle size distribution was measured using dynamic light scattering using a Zetasizer Nano ZSP (Malvern Instruments) to ensure that a final consistent size of between 30 and 40 nm was obtained (Fig. S9). Lipid concentration used is the monomer equivalent concentration.

### Measurement of aggregation kinetics using lipid vesicles

Solutions containing αS (100 μM), DMPS vesicles (200 μM), ThT (50 μM), and sodium azide (0.01%) in 20 mM sodium phosphate buffer (pH 6.5) were prepared in a half-area 96-well nonbinding plate (Corning; code: 3881), sealed with aluminum Thermowell sealing tape (Corning; code: 6570), and incubated in a CLARIOstar plate reader (BMG Labtech) for up to 400 h at 30 °C under quiescent conditions. Samples had a volume of 100 μl. Readings were taken at 1200 s intervals; λexcitation = 440 to 10 nm and λemission = 480 to 10 nm, gain = 800, and focal height = 4.9 mm. Each experiment was carried out in triplicate, and error bars represent standard error.

Periodically, the plate was removed from the plate reader and incubated at 30 °C for up to 200 h. This is especially the case for the longest incubation up to 600 h.

### Measurement of fibril seeding potential

Aggregation kinetics were recorded as aforementioned for the E46K mutant αS with nine technical replicates of each, in the presence and absence of DMPS vesicles. At time points 43, 200, and 455 h, three of the replicates were removed and combined (300 μl total). About 5 μl of these samples was used for TEM analysis. The remaining solutions were centrifuged at 14,000*g* on a desktop centrifuge for 30 min, and the supernatant containing soluble protein was removed leaving only the insoluble fibril pellet. To this pellet, 350 μl of fresh solutions containing E46K αS (100 μM), ThT (50 μM), and sodium azide (0.01%) in 20 mM sodium phosphate buffer (pH 6.5) were added. The solution was mixed by vortexing and freeze–thawed five times in liquid nitrogen and a sonicating water bath to break the fibrils into fibril seeds. These solutions were then added to a half-area 96-well nonbinding plate in 3 × 100 μl technical replicates, sealed with aluminum Thermowell sealing tape, and incubated again in a CLARIOstar plate reader for up to 175 h at 37 °C under quiescent conditions. The resulting samples were again analyzed by TEM to determine the change in fibril morphology.

### CD spectroscopy

Far-UV CD spectra were recorded using a Chirascan V100 (Applied Photophysics) with a Peltier thermally controlled cuvette holder at 30 °C. Quartz cuvettes with a 1 mm path length were used, and CD spectra were obtained by averaging three individual spectra recorded with a 1 nm bandwidth, measuring between 280 and 190 nm. Each sample was blanked against the buffer used in the aggregation assay.

Samples prepared for the measurement of aggregation kinetics using lipid vesicles were diluted 10-fold in order to have a final αS concentration of 10 μM.

Samples prepared for the measurement of binding to DMPS lipid vesicles had a total volume of 200 μl, with a constant αS concentration of 10 μM. DMPS lipid vesicle concentration was either 0, 10, 50, 100, 250, 500, or 1000 μM. The samples were incubated at 30 °C for 1 h before the spectra were recorded. CD spectra were fitted using DichroWeb, using analysis program CDSSTR and reference set 7 (77, 78).

### TEM

αS samples from the end point of the aggregation kinetics were collected. About 5 μl of these samples were put onto on glow-discharged Formvar/carbon-coated, 200 mesh, copper grids for 1 min. The samples were dried with filter paper and washed twice with MilliQ water for 1 s, each time removed with filter paper. The sample was stained by incubating the grids with 5 μl Uranyl Acetate Zero (Agar Scientific) for 30 s, followed by removal of the excess stain with filter paper. The grids were left to air-dry for 2 h. The samples were imaged using a TEM Jeol 2100 Plus (JEOL), operating at an accelerating voltage of 200 kV. Multiple grids were screened to obtain representative images of the samples. Fibril morphology was analyzed using ImageJ (National Institutes of Health) (79).

### Neuroblastoma cell culture

Human neuroblastoma cell line SH-SY5Y (ECACC [European Collection of Authenticated Cell Cultures]; catalog no.: 94030304) was purchased from Public Health England’s ECACC. Unless otherwise stated, all cell culture consumables were purchased from Thermo Fisher. Cells were cultured in Dulbecco’s modified Eagle’s medium (DMEM)/F-12 media with phenol red and without Hepes and l-glutamine. DMEM/F12 was supplemented with fetal bovine serum (10%), l-glutamine (2 mM), and nonessential amino acids (5%); with penicillin (100 IU) and streptomycin (100 mg/ml) (P/S; Corning). The culture was maintained in an incubator at 37 °C, 5% CO_2_, and saturated humidity until about 80% confluency was reached, for a maximum of 20 passages. For toxicity assays, the stock culture was seeded in 24-well plates and grown at 37 °C, 5% CO_2_, and saturated humidity for 24 h to reach 60% confluency prior to differentiation. Cells were seeded at 1 × 10^6^ cells/ml.

### SH-SY5Y differentiation

The differentiation of SH-SY5Y cells was carried out based on the method from Förster *et al.* (80) using two steps and phase 1 and phase 2 media (80). Phase 1 medium (DMEM containing l-glutamine [4 mM] and glucose [25 mM], P/S [1%] and no sodium pyruvate; retinoic acid [10 μM, Merck] added just prior to addition to cells) was added to the cells on days *in vitro* (DIV) 1. Phase 2 medium (Neurobasal A medium without phenol red, l-glutamine [1%], N-2 supplement [1%], and P/S [1%]; human brain-derived neurotrophic factor [50 ng/ml, Merck] added shortly before adding to the cells) was added to the cells on DIV 5. Cells were left to differentiate until DIV 8 at 37 °C, 5% CO_2_, and saturated humidity.

### PI/Hoechst assay

αS variants (100 μM) were incubated either with DMPS vesicles (200 μM) or without DMPS vesicles as a control sample in 20 mM sodium phosphate buffer, pH 6.5, for the indicated length of time at 30 °C. Aliquots of the incubated samples were added to the media of differentiated SH-SY5Y neuroblastoma cell cultures to a final concentration of 20 μM αS in triplicate. The plate was incubated for 48 h at 37 °C, 5% CO_2_, and saturated humidity. Cell viability was assessed by PI/Hoechst 33342 assay. Briefly, the cell growth media were removed and replaced with equivalent volume of growth media containing 10 μl/ml Hoechst solution and incubated at 37 °C, 5% CO_2_, and saturated humidity for 15 min. The Hoechst solution was then removed and replaced with PI (5 μl/1 ml) and incubated at 37 °C, 5% CO_2_, and saturated humidity for 15 min. The media were removed, and the wells were imaged in triplicate under both *blue* (Hoechst, live cells) and *red* fluorescence (PI, *red*). The number of cells corresponding to each fluorescence was counted, and the cell viability was determined by normalizing the live (Hoechst, *blue*)/dead (PI, *red*) ratio to the buffer control.

## Data availability

Data used in this study are located in the article, the supporting information, or are available upon request.

## Supporting information

This article contains supporting information (106).

## Conflict of interest

J. M. M. is an advisor to Sapience Therapeutics. All other authors declare that they have no conflicts of interest with the contents of this article.
