# Supplementary material for: Anionic lipid vesicles have differential effects on the aggregation of early onset-associated α-synuclein missense mutants
Source: J Biol Chem. 2022 Oct 5;298(12):102565. doi: 10.1016/j.jbc.2022.102565 (PMC9694135; doi:10.1016/j.jbc.2022.102565)
Supplement: Supplemental information [file mmc1.doc]

# Supporting Information

## CD analysis

CD spectra were fitted using Dichroweb, using analysis program CDSSTR and reference set 7. Total β-sheet content is given as the sum of the Strand 1 and Strand 2 values*.*

**
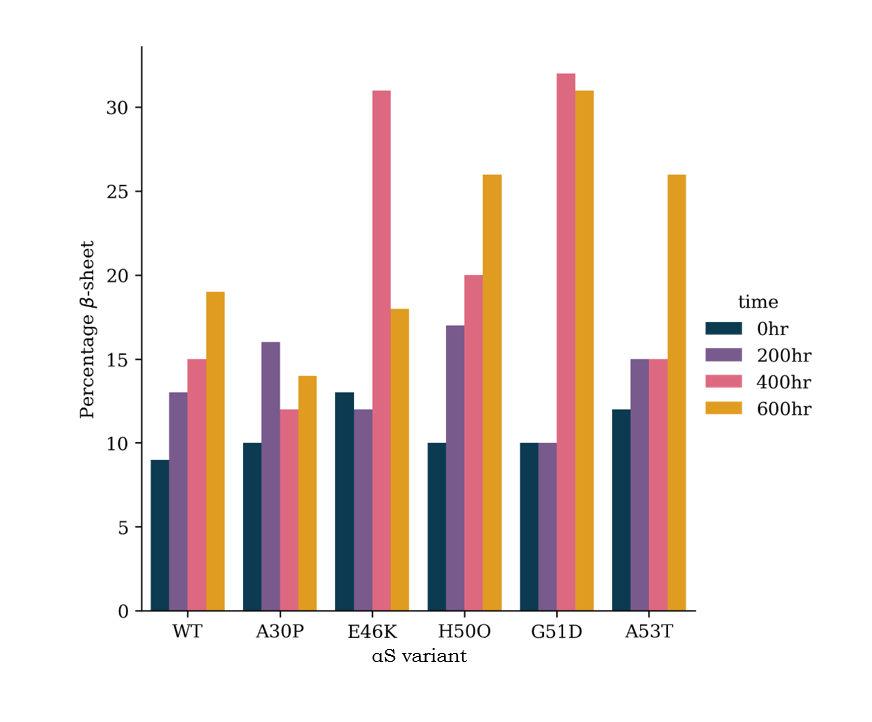
**

**Fig S1:** CD spectra were fitted to deconvolute the spectra into secondary structure compositions. Shown is the % β-sheet at 0hr, 200 hr, 400 hr and 600 hr for each of the 6 α-synuclein variants.

## ThT replicates

ThT assays were repeated on several occasions, with different batches/ preparations of protein. Shown in Fig S2 is the data for the biological repeats for E46K and G51D showing that the data contained in our paper is highly reproducible for G51D (D-F), with more variation seen in the aggregation profile of E46K (A-C), however the overall trend can be seen throughout the repeats.


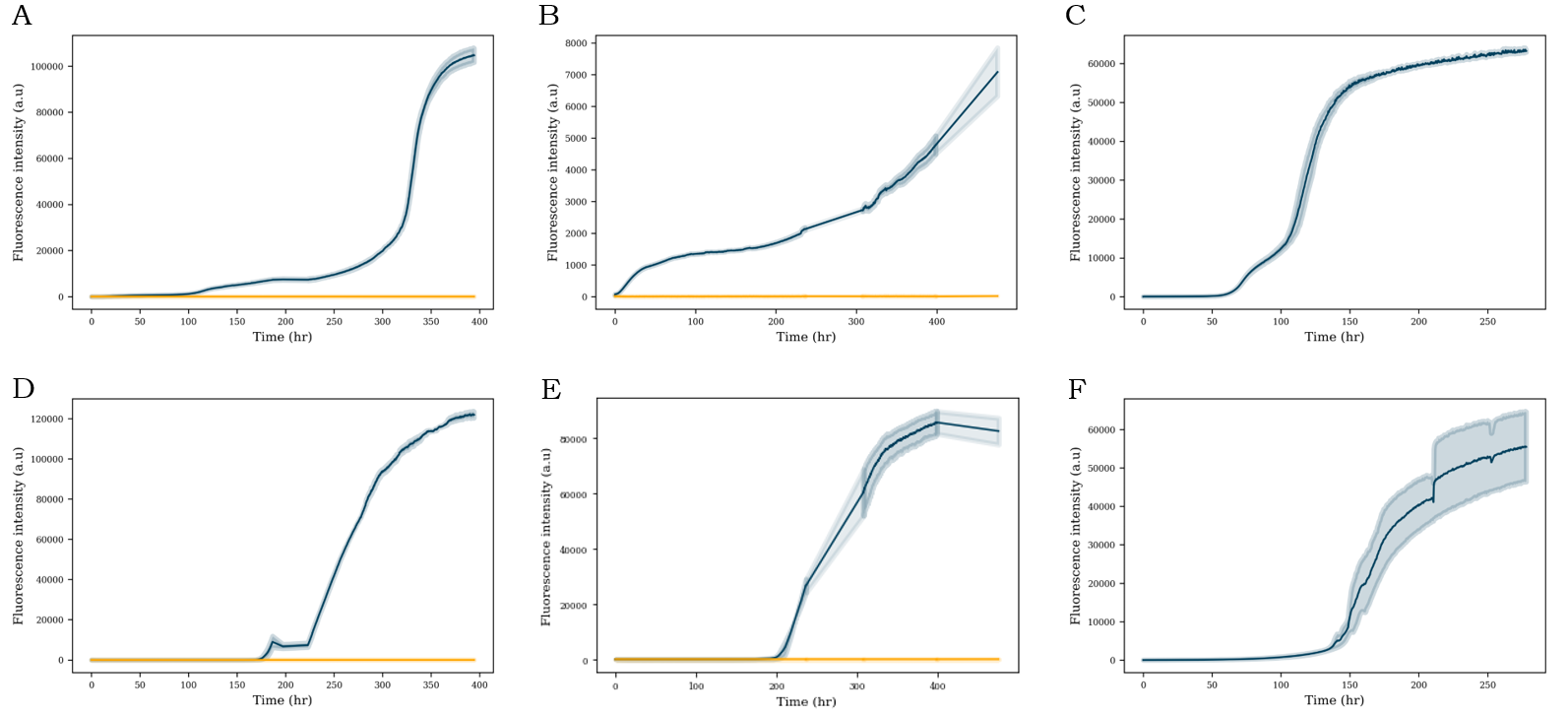


**Fig S2:** **ThT replicates of E46K and G51D.** A-C) E46K ThT aggregation data comparison between different assays, using different αS preparations. All conditions are the same (αS (100 μM) and DMPS (200 μM) however assay C was done in a 384-well plate compared to A and B in 96-well plates. This might explain some of the difference in the kinetics, however the trend in aggregation profile is very similar to A. D-F) G51D ThT aggregation data comparison between different assays, using different αS preparations. All conditions are the same (αS (100 μM) and DMPS (200 μM) however assay F was done in a 384-well plate compared to D and E in 96-well plates. All show a very similar pattern in aggregation profile and kinetics. All data shown in the average of 3 technical repeats, with the shading indicating the standard error.


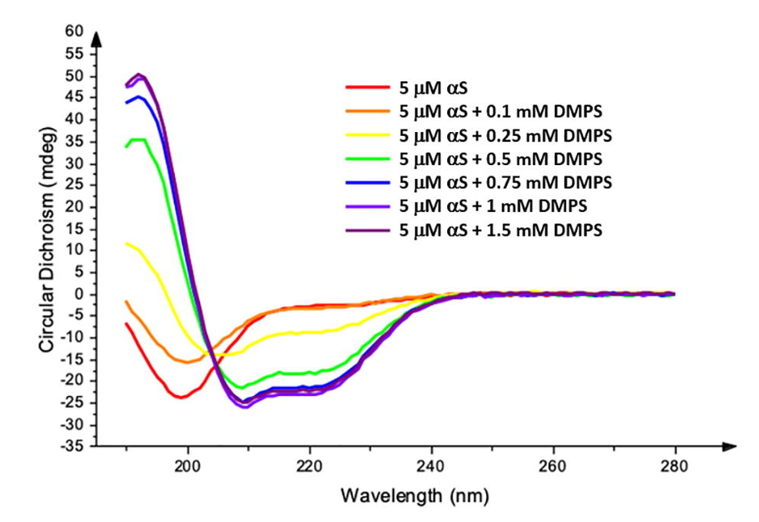


**Fig S3:** **Circular Dichroism studies in the presence of lipid vesicles, taken from Meade et al., demonstrates αS conformational change to α-helical conformation is proportional to DMPS SUV concentration SUVs** In isolation αS (5 μM), in 20mM phosphate buffer (pH 6.5) exists as a random coil. The αS conformation shifts towards an α-helical structure with increasing concentration of DMPS SUVs (0.1 to 1.5 mM). The αS is observed to reach maximal α-helical conformation at a ratio of 200:1 DMPS:αS.


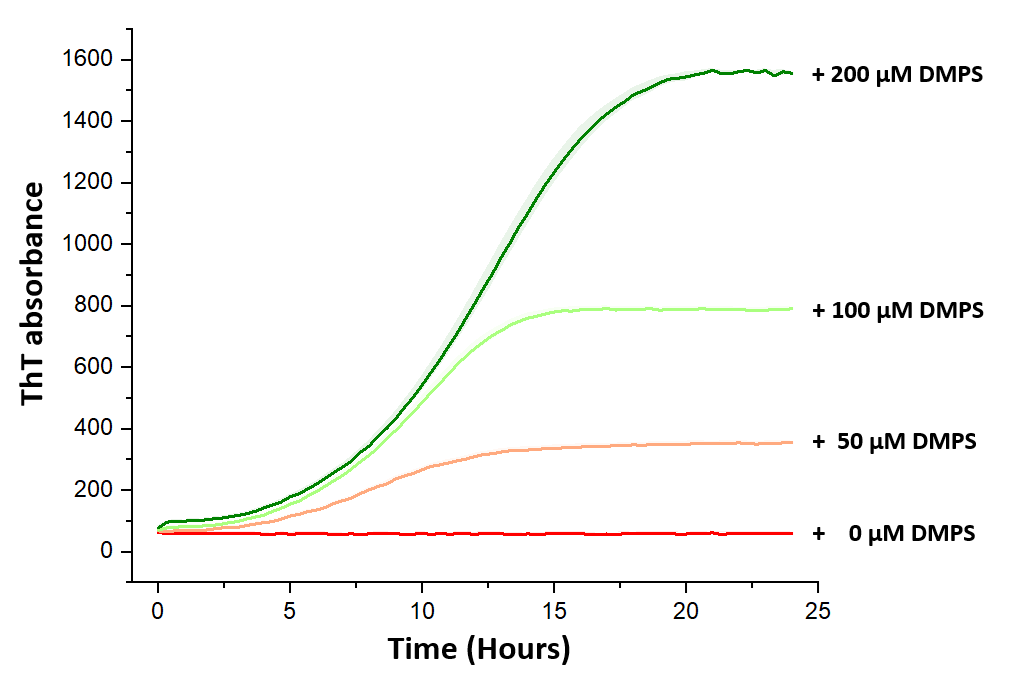


**Fig S4:** **ThT monitored αS aggregation is proportional to the concentration of DMPS SUVs.** ThT fluorescence intensity when 100 μM αS is incubated with 0 μM, 200 μM , or 400 μM DMPS vesicles and 50 μM Thioflavin T in 20 mM phosphate buffer (pH 6.5) under quiescent conditions at 30 °C.


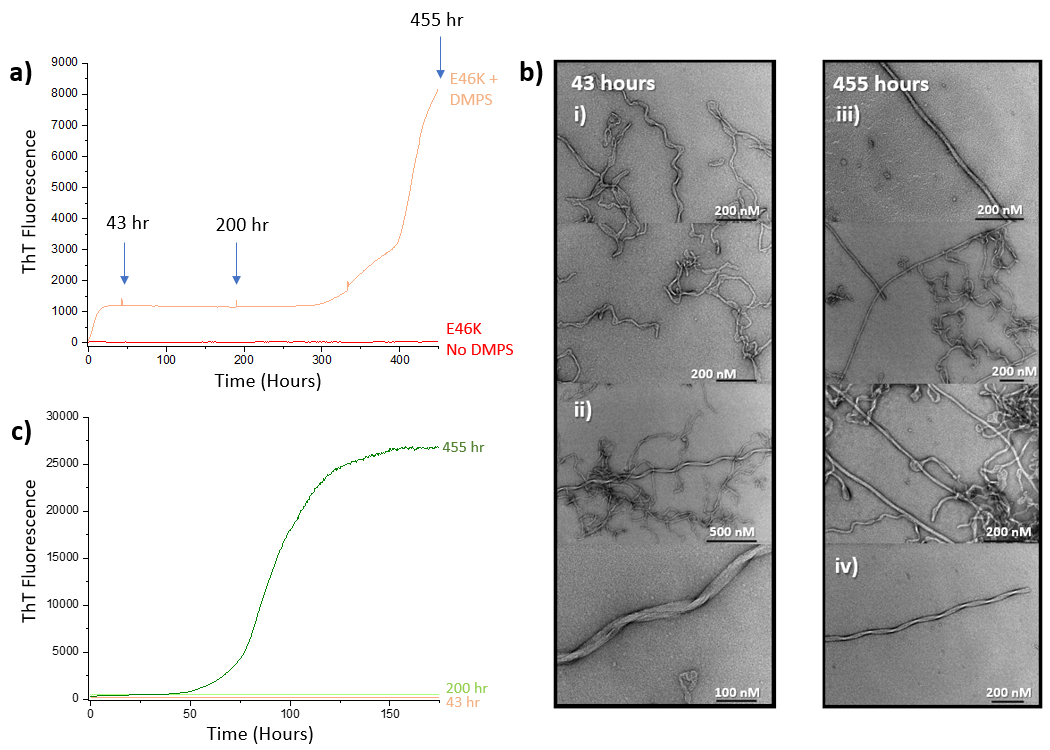


**Fig S5: Seeding potential of E46K fibril polymorphs. a)** ThT fluorescence intensity when 100 μM E46K αS is incubated with 200 μM DMPS vesicles (orange) and without vesicles (red) and 50 μM Thioflavin T in 20 mM phosphate buffer (pH 6.5) under quiescent conditions at 30 °C for 455 hours. Samples were taken at 43hr (after the first ThT fluorescence transition), 200 hours, and 455 hours (after the second transition ThT fluorescence transition). **b)** TEM images of the fibrils formed at 43 hours (i), and (ii) and at 455 hours (iii) and (iv). The fibrils at 43 hours presented a wave like morphology (i) and the larger helical polymorphs (ii). The fibrils observed 455 hours also presented a wave like morphology (iii) and the larger helical polymorphs (iv), but in addition there was the presence of straighter fibrils typically reported (iii). **c)** The samples collected at 43 hours, 200 hours and 455 hours from the first aggregation were centrifuged to harvest the fibril. These were mixed with fresh monomeric αS aggregation solution, and freeze thawed to produce fibril seeds. Only seeds collected at the 455 hr time point (dark green), after the second ThT transition, seeded the growth of new fibrils. In contrast, seeds collected after the first transition, 43 hours (orange) and 200 hours (light green), did not seed the growth of new fibrils.

**SDS-PAGE of soluble fraction at ThT Plateau (40 hr):**

Aggregation kinetics was performed as previously outlined. Briefly, solutions containing α-synuclein (wt, A30P, E46K, H50Q, G51D and A53T) (100 µM), DMPS vesicles (200 µM), ThT (50 µM) and sodium azide (0.01%) in 20 mM sodium phosphate buffer (pH 6.5) were prepared, in triplicate, in a half-area 96-well nonbinding plate (Corning 3881), sealed with aluminium Thermowell sealing tape (Corning 6570), and incubated in a CLARIOstar plate reader (BMG Labtech). After 40 hr, when the initial aggregation step was complete, the triplicate samples were collected and centrifuged (Eppendorf) for 1 hr at 14,500 rpm to pellet any insoluble aggregates. The supernatant was then analysed by SDS-PAGE gel electrophoresis, and the SDS resistant bands compared.


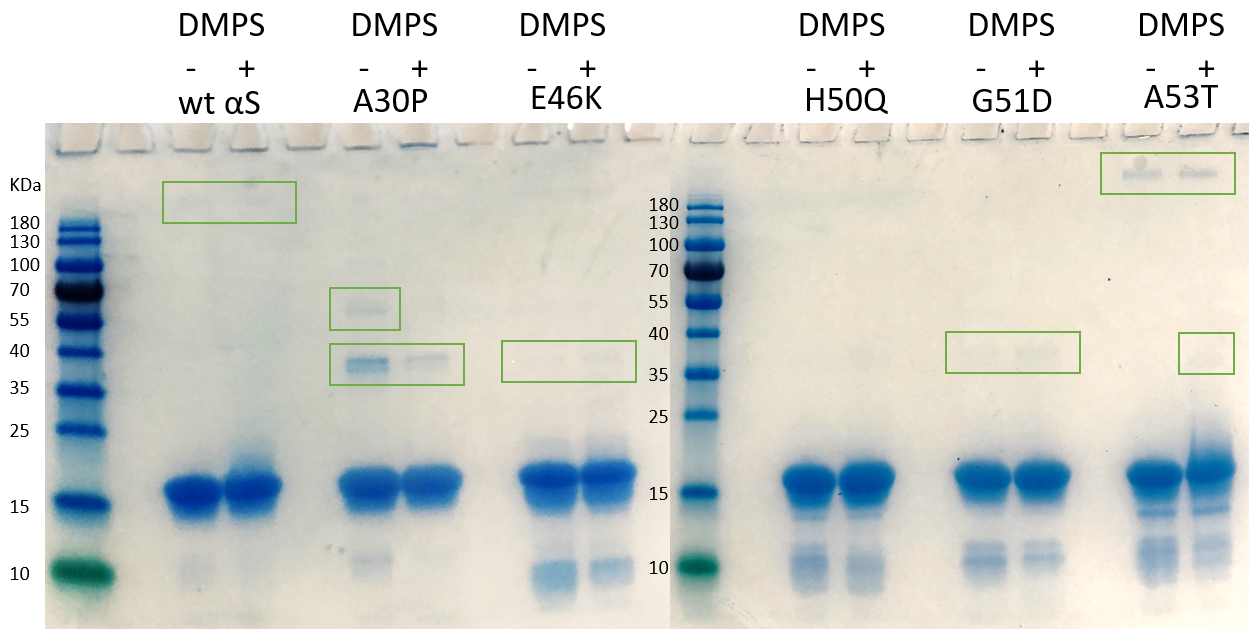


**Fig S6:** **SDS-PAGE of lipid induced aggregation of early onset αS mutants after 40 hr**. The SDS resistant oligomeric bands are observed for different αS mutants (highlighted in green boxes for clarity) after the initial stage of aggregation (40 hr). The different αS mutants show distinct oligomeric distributions in the absence and presence of lipid vesicles compared to wt αS. All present a monomeric band, as expected, at ~15 kDa. All mutants, except for H50Q, present an oligomer band between 35-40 kDa, which is not observed in the wt αS sample, which only presents a faint high-n order oligomer, both in the absence and presence of DMPS vesicles. An additional band is observed in the A30P mutant in the absence of lipids at ~60 kDa, and distinct high-n order oligomers are observed in the G51D mutant both in the absence and presence of the DMPS vesicles.
